# An online national quality assessment survey of prostate MRI reading: interreader variability in prostate volume measurement and PI-RADS classification

**DOI:** 10.1016/j.ejro.2024.100625

**Published:** 2024-12-12

**Authors:** Jonas Wallström, Erik Thimansson, Jim Andersson, Mathias Karlsson, Sophia Zackrisson, Ola Bratt, Fredrik Jäderling

**Affiliations:** a Department of Radiology, Institute of Clinical Sciences, Sahlgrenska Academy, University of Gothenburg, Sweden; b Department of Radiology, Sahlgrenska University Hospital, Gothenburg, Sweden; c Department of Translational Medicine, Faculty of Medicine, Lund University, Sweden; d Department of Radiology, Helsingborg Hospital, Helsingborg, Sweden; e Equalis AB, Uppsala, Sweden; f Department of Medical Sciences, Clinical Chemistry, Uppsala University, Sweden; g Department of Imaging and Physiology, Skåne University Hospital, Malmö, Sweden; h Department of Urology, Institute of Clinical Sciences, Sahlgrenska Academy, University of Gothenburg, Sweden; i Department of Urology, Sahlgrenska University Hospital, Gothenburg, Sweden; j Institution of Molecular Medicine and Surgery (MMK), Karolinska Institutet, Stockholm, Sweden; k Department of Radiology, Capio S:t Görans Hospital, Stockholm, Sweden

**Keywords:** Magnetic resonance imaging, Prostatic Neoplasms, Observer Variation, Radiology, Training

## Abstract

**Background:**

High-quality assessment of prostate MRI is fundamental in both clinical practice and screening. There is a lack of national level data on variability in prostate volume measurement and PI-RADS assessment. Methods of quality assurance need to be developed.

**Methods:**

All Swedish radiology departments were invited to participate in an external quality assurance of prostate MRI reading. Ten prostate MRI cases were selected by an expert panel to reflect common findings. Readers measured whole gland volume (ellipsoid formula method) and assigned a PI-RADS score in a web-based PACS with full clinical functionality. Expert consensus was used as reference standard. Descriptive statistics were used to show the distribution of volume measurements and PSA density. Reader agreement was assessed using percentages and kappa scores. A feedback document was sent to all participants upon completion of the quality assurance program.

**Results:**

Forty-three radiologists representing 17 departments read at least 7 out of 10 cases. The median difference in prostate volume assessment compared to the reference volume for the 10 cases ranged from −23 mL to + 6 mL. Per case agreement ranged from 33 % to 86 % for the assigned PI-RADS score and from 35 % to 98 % for PI-RADS 1–3 versus PI-RADS 4–5. Interreader agreement was moderate with a median kappa score of 0.53 (IQR 0.48–0.62).

**Conclusion:**

This online model for national quality assurance programs was feasible. Rather large per-case reader variations in prostate volume assessment and PI-RADS scoring were shown. To reduce variability in clinical practice, systematic interreader comparisons should be encouraged.

## 1 Introduction

With the paradigm shift to ´MRI first´ , prostate magnetic resonance imaging (MRI) became a cornerstone in the diagnostic pathway of prostate cancer [1], [2], [3]. MRI is now used to select patients for biopsy. Prostate MRI is assessed by using the Prostate Imaging-Reporting and Data System (PI-RADS) classification, in which lesions are assigned a score from 1 to 5 indicating an increasing likelihood of clinically significant cancer[4], [5]. A pre-biopsy MRI involves prostate volume measurement for the calculation of PSA density (PSAD), which is the ratio between the serum PSA value and the prostate volume. PSAD better predicts clinically significant cancer than the PSA value alone and is recommended as an adjunct to the PI-RADS score for risk stratifying patients before biopsy [6].

One of the important limitations of PI-RADS is interreader variability [7]. This has led to a discussion about how to optimize training of radiologists [8] including initiatives for certification and regular training courses, such as the European Society of Urogenital Radiology (ESUR) prostate MRI course. These initiatives aim to provide the radiologist with the means to detect clinically relevant tumors and avoid unnecessary biopsies that may lead to overdiagnosis and complications.

The aim of this national external quality assurance of prostate MRI assessment was to review the current quality of prostate MRI readings in Sweden as a basis for understanding how to design future quality assessments. We intend to use the results to establish a long-term educational initiative to ensure consistent high quality radiological MRI assessments, a prerequisite for a successful implementation of organized prostate cancer testing and future screening programs for prostate cancer.

## 2 Methods

### 2.1 Survey design, case selection, and expert group

The external quality assurance program, held between the 26th of April and the 21st of May 2023, was conducted by representatives for the Swedish OPT program and the non-profit company Equalis, which is owned by the Swedish Association of Local Authorities and Regions and the Swedish Society of Medicine. Equalis focuses on ensuring reliable and comparable results for laboratory medicine, medical imaging, and functional medicine [9].

Three experts formed a consensus group, all sub-specialized in prostate MRI with responsibility for regular multidisciplinary team meetings and a high level of experience from histological feedback. The experts were chosen from different sites.

The test cases were prostate MRI scans from clinical practice in the consensus group members´ departments. Thus, cases were selected from the imaging databases of two tertiary referral centers and one university hospital. In total, six scanners from three different vendors were used (Table S2). All scanners except one had a field strength of 3 T. The imaging protocols were standardized bi-parametric protocols [10], [11], [12] as recommended by Swedish national guidelines. The technical specifications were in accordance with the PI-RADSv2.1 document [4]. In brief, T2-weighted images, covering the prostate with a slice thickness of 3 mm, were acquired in three planes. Diffusion weighted images were acquired in the axial plane. An extrapolated high b-value of 1500 s/mm^2^ was used. The ADC map was calculated using a low b-value of ≤ 100 s/mm^2^ and a high b-value of 800–1000 s/mm^2^.

Ten cases were selected for feasibility reasons to represent a range of PI-RADS scores and prostate volumes. The consensus group reviewed the ten test cases and agreed on prostate volume, lesion localization, and PI-RADS scores. For eight of the ten cases, prostate biopsy results were available, for two also subsequent radical prostatectomy histology. The test cases were assessed for image quality and had a PI-QUALv2 score of ≥ 2 [13]. All cases were anonymized before review by the participants. Characteristics and expert consensus for the ten cases are presented in Table 1

**Table 1:** Characteristics of cases selected by the expert consensus and histological biopsy outcomes, if available.

Case	PSA (ng/mL)	Expert consensus prostate volume (mL)	Expert consensus PSA Density (ng/mL^2^)	Expert consensus PI-RADS (1–5)	Lesion location zone (PZ/TZ)	Biopsy (TBx/SBx/no)	Gleason Grade Group (ISUP)
1	12	77	0.16	2	-	SBx	benign
2	8	143	0.06	3	TZ	no	N.A.
3	7.1	87	0.08	5	PZ	TBx	2
4	3.4	24	0.14	2	-	SBx*	benign
5	2.6	34	0.08	3	PZ	TBx* *	2
6	5.6	52	0.11	4	PZ	TBX	2
7	4	57	0.07	2	-	no	N.A.
8	3.9	25	0.16	5	TZ	TBx	2
9	4	63	0.06	3	TZ	TBx* *	benign
10	9	55	0.16	3	PZ	TBx	benign

PSA=Prostate specific antigen; PSAD=PSA Density; PI-RADS=Prostate Imaging-Reporting and Data System; PZ=Peripheral Zone; TZ=Transition Zone; SBx=Systematic biopsy; TBx=Targeted biopsy; ISUP=International society of urological pathology.

* Systematic biopsy based on PSAD ≥ 0.15 ng/mL^2^. Re-evaluated volume by the expert consensus yielded a PSAD < 0.15 ng/mL^2^.

** Biopsied according to STHLM-3 test algorithm.

At the time of the study, national guidelines recommended a biopsy for PI-RADS 4–5 and for PI-RADS 3 if prostate specific antigen (PSA) density was ≥ 0.15 ng/mL^2^.

### 2.2 Survey distribution, participant reporting, and feedback

**Fig. 1:**
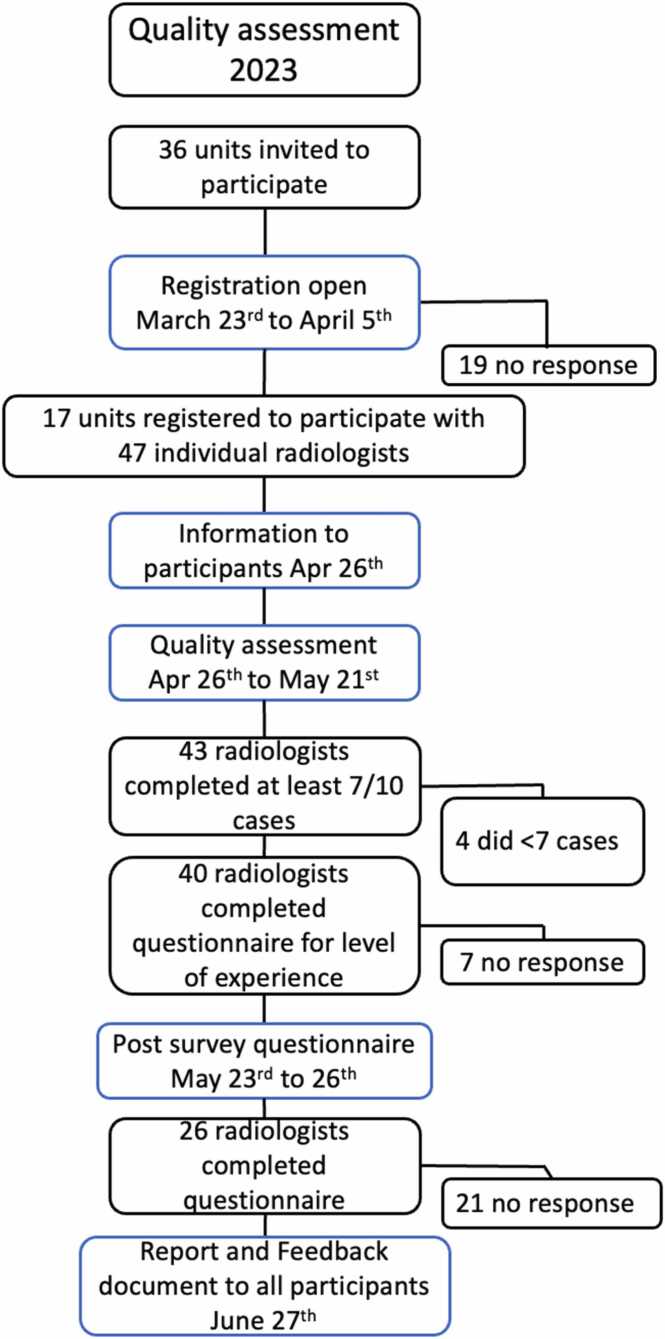
Quality assessment survey flowchart.

All radiology departments in Sweden were invited in 2023 to participate in the survey free of charge. Fig. 1 outlines a timeline for the survey design, from invitation to post-survey questionnaire, with the number of participants fulfilling each part. MRI readings had to be carried out individually by the participants. All participants were anonymous to the survey organizers.

Before case reviews, the participants submitted information about level of specialization, number of prostate MRIs reported annually, experience of histology feedback (biopsy and/or radical prostatectomy specimens), and experience of multi-disciplinary team conferences.

The participants then reviewed the ten cases on Collective Minds (Collective Minds Radiology, Danderyd, Sweden) a web based PACS (Picture and Archiving and Communication System) with full clinical functionality including possibilities to zoom, pan, measure, and so on. The cases included full image stacks for review, including both T2-weighted images (in axial, coronal, and sagittal planes) and high b-value (b1500) diffusion weighted images with corresponding ADC-map (in axial plane). A specific screen resolution for reading cases was not defined. The participants’ interpretations were registered on a web-based template at the Equalis server.

The participants were informed about the patient´s age and PSA values, but no other clinical information was made available.

The participants were also asked to measure the width, height, and depth of the prostate and to calculate the volume according to the ellipsoid formula (width x height x depth x π/6).

After completing the quality assessment, a post-survey questionnaire was sent to all participants to assess their general impression of the survey, as well as a feedback document with a description of imaging findings, expert PI-RADS scores, and histological correlation for each case (Supplement 1).

### 2.3 Ethical considerations

This study was an external quality assurance of prostate MRI assessment without any personal data. All images and data were anonymized to Equalis and the expert group. Hence, the study did not require ethical review according to the Swedish Research Ethics Act (2003:460).

### 2.4 Statistical methods

The expert consensus was used as reference standard. Distributions of prostate volume measurement differences were visualized with box plots. The reported prostate volumes were used to calculate the PSA density (ng/mL^2^) for each participant and compared to consensus. PI-RADS agreement for participant assessment versus reference standard was assessed using percentages and Cohen’s weighted kappa with 95 % confidence intervals. Kappa values are usually interpreted as follows: 0–0.20 is slight agreement, 0.21–0.40 fair, 0.41–0.60 moderate, 0.61–0.80 substantial, and 0.81–1 almost perfect agreement. Statistical analyses were performed with SPSS v29 and R version 4.2.2.

## 3 Results

Of 36 invited radiology departments 17 participated (public, private, and teleradiological). Forty-three participating radiologists; 36 specialists, 2 residents and 5 unspecified reported at least 7 of the 10 cases. Their self-reported average number of cases read per year was 260 (range 5–1023, median 167, interquartile range 85–400). Nine radiologists had experience from more than 50 cases with histological feedback from targeted biopsy, 12 had 20–50 cases with feedback, 19 had less than 20 cases, and 3 did not respond. Thirteen of the radiologists regularly lead a prostate cancer multi-disciplinary team conference.

**Fig. 2:**
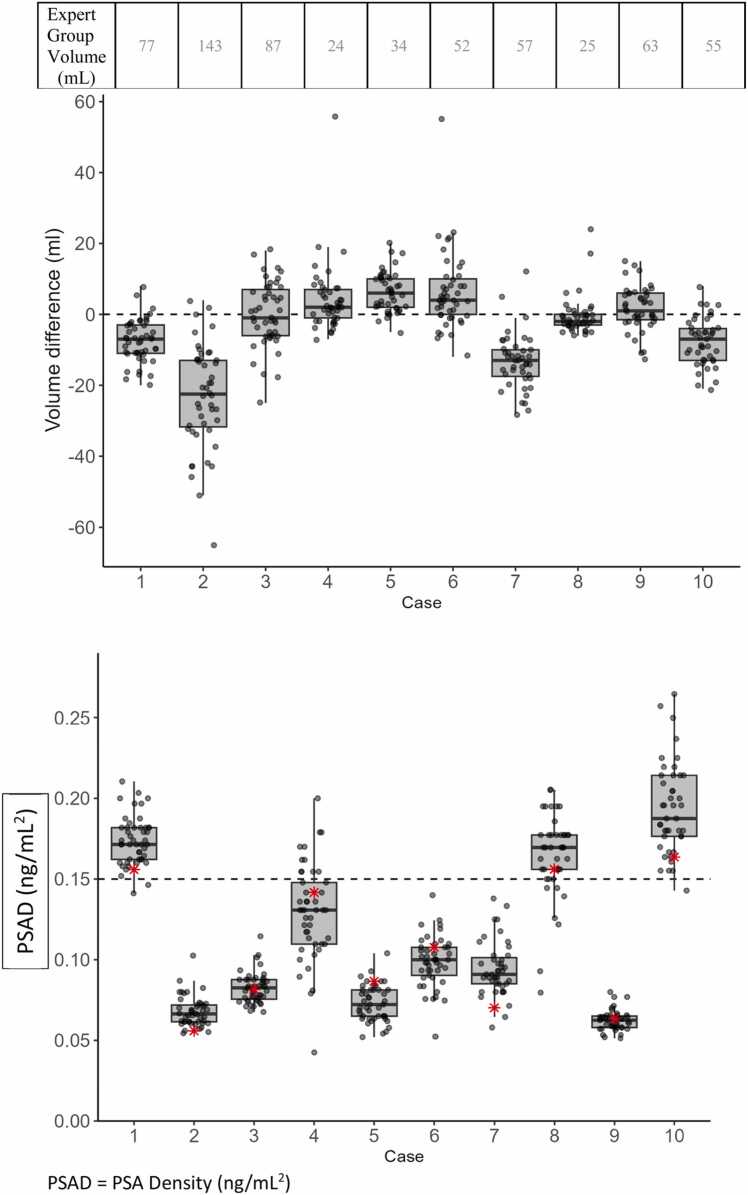
**a**. Box plots showing individual and average differences in calculated prostate volume with expert consensus as reference standard (zero line). The reference volume for each case is shown in the top panel. Fig. 2**b**. Box plots showing individual and average differences in PSA density compared to expert consensus reference. The expert consensus PSAD is marked with a red asterisk for each case. The dotted reference line (0.15) marks the OPT threshold for biopsy indication. PSAD = PSA Density (ng/mL^2^).

A total of 447 case readings were completed. Fig. 2**a** illustrates the interreader variation in volume assessment with the expert consensus measurement as reference. The average difference in prostate volume assessment per case ranged from −23 to + 6 mL compared to consensus. The inter-individual differences were more pronounced, for example case 6 with measurements ranging from 40 to 107 mL compared to the 52 mL expert consensus volume.

Fig. 2**b** illustrates the interreader variation in the resulting PSAD with the expert consensus as reference and the biopsy threshold of 0.15 ng/mL^2^ (according to Swedish national guidelines at the time of the study) marked by a dotted line. The average PSAD difference ranged from −0.2–0.3 ng/mL^2^ compared to consensus. Interreader differences ranged from −0.11 to + 0.10 ng/mL^2^ per case. PSAD threshold agreement was overall high but somewhat lower in two cases with small prostate volumes (24 mL and 25 mL) where 35/45 (78 %) and 39/44 (89 %) readers agreed.

The agreement of PI-RADS scoring per case (participants versus expert consensus) is shown in Table 2. Per case agreement ranged from 33 % to 86 % for the assigned PI-RADS score and from 35 % to 98 % for PI-RADS 1–3 versus PI-RADS 4–5. The lowest agreement for the assigned PI-RADS score was found in cases 2 (PI-RADS 3), 7 (PI-RADS 2), 9 (PI-RADS 3), and 10 (PI-RADS 3): 33 %, 47 %, 36 %, and 33 % respectively. Considering the agreement for PI-RADS 1–4 versus 4–5 the lowest agreement was found in cases 9 and 10 (Fig. 4): 61 %, and 35 % respectively.

**Table 2:** Distribution of PI-RADS scores and percentages of agreement.

Case	PI-RADS score by the expert consensus (1–5)	PI-RADS score distribution for the participants (1–5)				Percentage of participants that agreed with expert consensus: same PI-RADS	Percentage of participants that agreed with expert consensus: PI-RADS ≤ 3 versus PI-RADS ≥ 4
		≤ 2	3	4	5		
1	2	37	6	4	0	37/47 = 79 %	43/47 = 91 %
2	3	19	15	8	4	15/46 = 33 %	34/46 = 74 %
3	5	2	5	10	29	29/46 = 63 %	39/46 = 85 %
4	2	30	14	1	0	30/45 = 67 %	44/45 = 98 %
5	3	15	23	6	0	23/44 = 52 %	38/44 = 86 %
6	4	0	1	33	11	33/45 = 73 %	44/45 = 98 %
7	2	20	15	8	0	20/43 = 47 %	35/43 = 81 %
8	5	0	3	3	38	38/44 = 86 %	41/44 = 93 %
9	3	11	16	15	2	16/44 = 36 %	27/44 = 61 %
10	3	1	14	9	19	14/43 = 33 %	15/43 = 35 %

PI-RADS=Prostate imaging-reporting and data system.

**Fig. 3:**
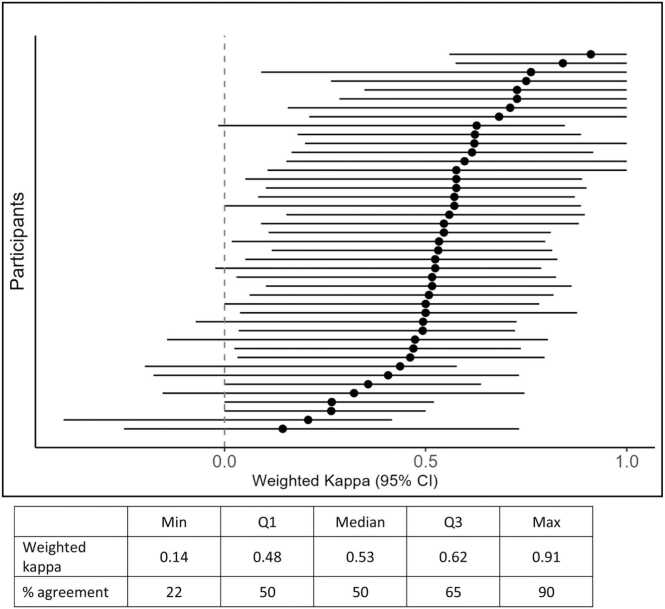
Inter-reader agreement for PI-RADS scoring. Weighted kappa scores for pairs of readers (participant number 1–43 versus expert consensus). Kappa scores interpreted as follows: 0–0.20 slight agreement, 0.21–0.40 fair, 0.41–0.60 moderate, 0.61–0.80 substantial, and 0.81–1 almost perfect agreement.

Fig. 3 shows weighted Cohens kappa for all individual readers versus expert consensus. The median weighted Cohens kappa for individual readers versus expert consensus was moderate: 0.53 (interquartile range 0.48–0.62). Six of 43 had slight to fair agreement, 25 had moderate agreement, 10 had substantial, and two almost perfect agreement.

Supplementary Table S1a**-c** show agreement versus level of experience. Out of the six readers with slight to fair agreement one did not disclose their level of experience. The remaining five were specialists that reported up to 599 cases per year and had received biopsy feedback from no more than 50 cases.

The post-assessment questionnaire was answered by 26 of radiologists. The average time to complete the quality assessment was 3 hours and 22 minutes. Sixteen were positive to the statement “I would recommend a colleague to participate in the Equalis prostate MRI quality assessment program”, 4 were neutral, and 6 were negative. Thirteen had a negative experience with the technical platform, 9 were neutral and 4 were positive.

## 4 Discussion

Ensuring high quality MRI reading is of paramount importance for the success of both clinical MRI pathways and future screening programs. This study shows that a broad panorama of radiologists from both public and private providers are willing to participate in national quality assessments of prostate MRI using a web-based PACS solution. In total 43 radiologists completed at least 7 out of 10 provided cases. According to the current Swedish clinical routine, the readers both assigned a PI-RADS score and measured the prostate volume, in contrast to previous inter-observer studies which involved PI-RADS assessment only [7], [14], [15], [16].

The current EAU guidelines suggest combining MRI findings with PSAD, preferably based on MRI obtained volumes, to inform biopsy decisions [6]. Thus, variations in volume assessment may affect the outcomes of diagnostic pathways. Our results show that overall, the agreement for a PSAD threshold of ≥ 0.15 ng/mL^2^ was high but that measurements were more varied in small prostates with subsequent consequences for the proportion of men with a biopsy indication. This is of importance as more men in the younger age span are expected to be tested in upcoming screening efforts [17].

In this study the ellipsoid formula was used for volume measurement, as recommended by the PI-RADS version 2.1 document. A previous study by Ghafoor et al., including four readers at the same institution, showed excellent agreement in prostate volume calculation using the ellipsoid formula [18]. Our study participants were from several different radiology departments, which may account for the greater variability observed in our study. How much variability can be reduced by providing feedback remains to be established in future assessments. The emerging deep learning based planimetry models will probably improve prostate volume assessment. These models save time and have robust performance also in multicenter settings, where they have achieved better agreement and precision than the ellipsoid formula and appear to provide a volume closer to the actual size of the prostate [19]. The present clinical guidelines are based on studies that calculated PSAD from prostate volume estimated via the ellipsoid formula (ultrasound and MRI) so the thresholds for biopsy may have to be adjusted if planimetry-based AI models are employed.

**Fig. 4:**
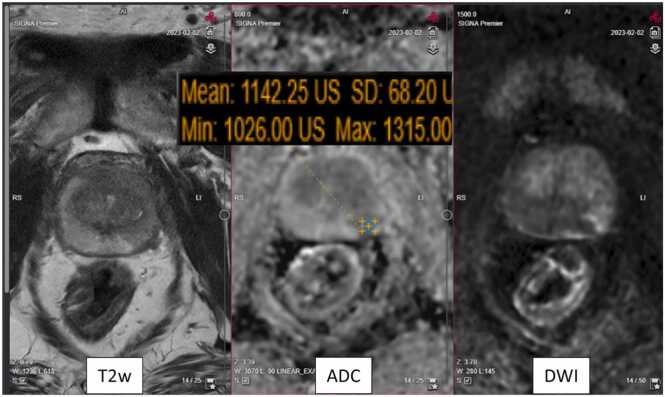
Case 10 with focal lesion in left dorsal peripheral zone. Low agreement between expert consensus (PI-RADS 3) and participants (PI-RADS 5; 19 participants and PI-RADS 4; 8 participants). Diffuse appearance of the lesion on T2 weighted transversal image (left) and only slight to moderately restricted diffusion with ADC-value 1142 × 10^−6^ mm^2^/s and DWI signal not markedly hyperintense (middle and right).

For the entire group of readers, the lowest PI-RADS score agreement was found in cases 9 and 10. Both these cases illustrate difficulties in assessing indeterminate lesions, i.e., PI-RADS score 3 [20]. For case 9 the expert group described the lesion as an area between nodules because of its irregular contours and therefore scored it as PI-RADS 3. This lesion was either under- or over-scored by most of the participants, which underlines the known challenges with transition zone lesions [7]. In case 10 (Fig. 4), only one third of the participants were concordant with the expert group assignment of PI-RADS 3. Most participants considered a large lesion in the left lateral segment as highly suspicious for significant cancer and scored it either as PI-RADS 5 (19 participants) or as PI-RADS 4 (9 participants) The expert consensus considered the lesion as indeterminate because of its diffuse appearance on T2 weighted images and only moderately restricted diffusion, DWI signal not markedly hyperintense [21]. Systematic and targeted biopsy were benign. According to the guidelines, a biopsy should have been performed regardless of MRI findings based on the high PSA-density. However, grading this case as PI-RADS 5 would likely have resulted in a repeat biopsy with additional risk of harm.

One key element to increase the accuracy in PI-RADS assessments, especially for indeterminate lesions, is follow-up of biopsy outcomes [22]. The ESUR/ESUI consensus statements on multiparametric MRI for the detection of clinically significant prostate cancer had a 100 % agreement for the statement that prostate radiologists should compare their performance with histopathological feedback [8]. To attain the intended benefits of the MRI pathway (reduced overdiagnosis of indolent cancer and increased diagnosis of significant cancer) radiologists must be confident in both ruling in and ruling out lesions. It is notable that although participants in this quality assessment reported an average of 260 cases per year, only one in five reported having correlated more than a total of 50 readings with biopsy outcomes. The National Prostate Cancer Register of Sweden (NPCR) has developed a national diagnostic IT platform that facilitates automatic biopsy histology feedback to the reporting radiologist [23]. Such feedback will aid the calibration process on an individual level and may reduce overcalling lesions and thereby overdiagnosis.

The overall agreement for PI-RADS scoring was moderate with half of the readers close to the median kappa value of 0.53, and one fourth of readers either clearly above or below the median. Although our results represent a broader panorama of readers and departments than most previous studies, they are in line with a meta-analysis by Park and co-workers that included 30 studies with 2–9 readers and reported a pooled kappa for PI-RADS 4–5 of 0.61 [16], [24]. For feasibility only ten cases were chosen in our study. Increasing the number of cases would have provided more reliable statistics but would likely have generated fewer responses. Considering the ambition to establish a baseline for future quality assessments, our results fall within the expected range. The feedback document sent to all the readers served as an educational effort to point out important features when assessing prostate MRI. Future studies should correlate level of experience with PI-RADS assessments and prostate volume calculations. Furthermore, the present study provides baseline data for future quality assessments.

Some interesting variations were observed in the group of low agreement readers. The readers with slight to fair agreement were all specialists reporting less than 600 cases per year. Considering that there were also readers with substantial to almost perfect agreement that had read the same number of cases per year (Supplementary Table 1) it appears that experience level alone does not always correlate with the degree of expertise. However, it has previously been shown that high volume expert readers have a significantly higher PPV for detecting cancer compared to non-specialized readers [25]. A steep learning curve for unexperienced readers has also been reported [26]. This raises the issue of the necessity of certification. The European Society of Radiology (ESR) has proposed certification of readers of prostate screening MRI [27]. Our results indicate that targeting educational efforts for low performance readers and increasing access to histological feedback may be areas to focus on in a future certification program.

Our quality assessment has some strengths and weaknesses.

A strength of our study is the great number of participating radiologists. We have not been able to find any previously published study of both volume measurements and PI-RADS assessments with so many participants. Our study demonstrates substantial variability between readers assessing the same case which could directly influence the patient-level clinical outcome.

A limitation is the restricted number of cases that could be included in this quality assessment. The case selection was made to cover common findings in both the peripheral zone and the transition zone with more indeterminate cases, in total 4 out 10 cases were PI-RADS 3, where a greater variability could be expected.

Another limitation is that although the number of participants was higher than in many other interreader studies it was too small for subgroup analysis, which would otherwise be of interest, especially to stratify results based on the level of experience. To increase the participation rate in a similar future survey, the participants’ questionnaire responses suggest a need to reduce the time required to complete the test (most participants used over 2 hours), improving the user-friendliness of PACS, and simplifying the templates for reported data.

## 5 Conclusions

An online prostate MRI quality assessment may be a feasible method for engaging radiologists from both private and public departments in quality assurance. Rather large per-case reader variations were shown in both volume assessment and PI-RADS scoring. To reduce variability in prostate MRI reading in clinical practice and in a screening setting, recurring systematic interreader comparisons should be encouraged.

## Ethical approval

Not applicable

This study was an external quality assurance of prostate MRI assessment without any personal data. All images and data were anonymized to Equalis and the expert group. Hence, the study did not require ethical review according to the Swedish Research Ethics Act (2003:460).

## Funding

None/Not applicable

## CRediT authorship contribution statement

**Mathias Karlsson:** Writing – review & editing, Validation, Supervision, Software, Resources, Project administration, Methodology, Data curation, Conceptualization. **Sophia Zackrisson:** Writing – review & editing, Supervision, Resources. **Fredrik Jäderling:** Writing – review & editing, Writing – original draft, Visualization, Validation, Supervision, Software, Resources, Project administration, Methodology, Investigation, Funding acquisition, Formal analysis, Data curation, Conceptualization. **Jim Andersson:** Writing – review & editing, Software, Resources, Project administration, Funding acquisition, Data curation, Conceptualization. **Ola Bratt:** Writing – review & editing, Supervision. **Erik Thimansson:** Writing – review & editing, Writing – original draft, Visualization, Validation, Supervision, Software, Resources, Project administration, Methodology, Investigation, Funding acquisition, Formal analysis, Data curation, Conceptualization. **Jonas Wallström:** Writing – review & editing, Writing – original draft, Visualization, Validation, Supervision, Software, Resources, Project administration, Methodology, Investigation, Funding acquisition, Formal analysis, Data curation, Conceptualization.

## Declaration of Competing Interest

The authors declare that they have no known competing financial interests or personal relationships that could have appeared to influence the work reported in this paper
