# Supplementary material for: An online national quality assessment survey of prostate MRI reading: interreader variability in prostate volume measurement and PI-RADS classification
Source: Eur J Radiol Open. 2024 Dec 12;14:100625. doi: 10.1016/j.ejro.2024.100625 (PMC11699621; doi:10.1016/j.ejro.2024.100625)
Supplement: Supplementary file 1 — Supplementary material [file mmc1.docx]

Supplementary: EJRO-D24-00230

**Supplementary table S2**

| Case | Scanner | Institution | Protocol | Sequences for review |
| --- | --- | --- | --- | --- |
| 1 | Philips Achieva dStream 3T | University Hospital 1 | bpMRI | T2 (axial, sagittal, coronal), DWI (b1500), and ADC-map |
| 2 | GE, Signa Premier, 3T | University Hospital 1 | bpMRI | T2 (axial, sagittal, coronal), DWI (b1500), and ADC-map |
| 3 | Siemens, Magnetom Vida 3T | Tertiary referral center 1 | bpMRI | T2 (axial, sagittal, coronal), DWI (b1500), and ADC-map |
| 4 | Siemens, Magnetom Aera, 1.5T | Tertiary referral center 1 | bpMRI | T2 (axial, sagittal, coronal), DWI (b1500), and ADC-map |
| 5 | GE, Signa Architect, 3T | Tertiary referral center 2 | bpMRI | T2 (axial, sagittal, coronal), DWI (b1500), and ADC-map T2 (axial, sagittal, coronal), DWI (b1500), and ADC-map |
| 6 | Siemens Magnetom Aera, 1,5T | Tertiary referral center 1 | bpMRI | T2 (axial, sagittal, coronal), DWI (b1500), and ADC-map |
| 7 | Philips, Ingenia, 3T | University Hospital 1 | bpMRI | T2 (axial, sagittal, coronal), DWI (b1500), and ADC-map |
| 8 | Siemens, Magnetom Vida, 3T | Tertiary referral center 1 | bpMRI | T2 (axial, sagittal, coronal), DWI (b1500), and ADC-map |
| 9 | GE, Signa Architect, 3T | Tertiary referral center 2 | bpMRI | T2 (axial, sagittal, coronal), DWI (b1500), and ADC-map |
| 10 | GE, Signa Premier, 3T | University Hospital 1 | bpMRI | T2 (axial, sagittal, coronal), DWI (b1500), and ADC-map |
