# Supplementary material for: An online national quality assessment survey of prostate MRI reading: interreader variability in prostate volume measurement and PI-RADS classification
Source: Eur J Radiol Open. 2024 Dec 12;14:100625. doi: 10.1016/j.ejro.2024.100625 (PMC11699621; doi:10.1016/j.ejro.2024.100625)
Supplement: Supplementary file 2 — Supplementary material [file mmc2.docx]

Supplements

Pdf1: Feedback document to participants

Supplementary tables S1 a–c. Tabulation of level of agreement versus level of experience.

Kappa scores interpreted as: 0–0.20 slight agreement, 0.21–0.40 fair, 0.41–0.60 moderate, 0.61–0.80 substantial, and 0.81–1 almost perfect agreement.

Table S1a: Level of specialisation versus agreement.

| Level of specialisation | Slight | Fair | Moderate | Substantial | Almost  perfect | Total |
| --- | --- | --- | --- | --- | --- | --- |
| Radiology residents | 0 | 0 | 1 | 1 | 0 | 2 |
| Radiology specialists | 1 | 4 | 21 | 8 | 2 | 36 |
| No reply | 0 | 1 | 3 | 1 | 0 | 5 |
| Total | 1 | 5 | 25 | 10 | 2 | 43 |

Table S1b: Cases read per year versus agreement.

| Cases read per year (n) | Slight | Fair | Moderate | Substantial | Almost perfect | Total |
| --- | --- | --- | --- | --- | --- | --- |
| 0–199 | 0 | 3 | 12 | 4 | 0 | 19 |
| 200-–399 | 1 | 0 | 4 | 1 | 1 | 7 |
| 400–599 | 0 | 1 | 4 | 2 | 0 | 7 |
| 600–799 | 0 | 0 | 2 | 1 | 0 | 3 |
| 800– | 0 | 0 | 0 | 0 | 1 | 1 |
| No reply | 0 | 1 | 3 | 2 | 0 | 6 |
| Total | 1 | 5 | 25 | 10 | 2 | 43 |

Table S1c: Number of cases with follow up of biopsy outcomes versus agreement.

| Biopsy feedback (n) | Slight | Fair | Moderate | Substantial | Almost perfect | Total |
| --- | --- | --- | --- | --- | --- | --- |
| 0–20 | 1 | 2 | 8 | 7 | 1 | 19 |
| 21–50 | 0 | 2 | 9 | 1 | 0 | 12 |
| >50 | 0 | 0 | 6 | 1 | 1 | 8 |
| No reply | 0 | 1 | 2 | 1 | 0 | 4 |
| Total | 1 | 5 | 25 | 10 | 2 | 43 |
