# Supplementary material for: An online national quality assessment survey of prostate MRI reading: interreader variability in prostate volume measurement and PI-RADS classification
Source: Eur J Radiol Open. 2024 Dec 12;14:100625. doi: 10.1016/j.ejro.2024.100625 (PMC11699621; doi:10.1016/j.ejro.2024.100625)
Supplement: Supplementary file 3 — Supplementary material [file mmc3.pdf]

EQUALIS – PROSTATE MRI

Case 1. Expected response: PI-RADS 2

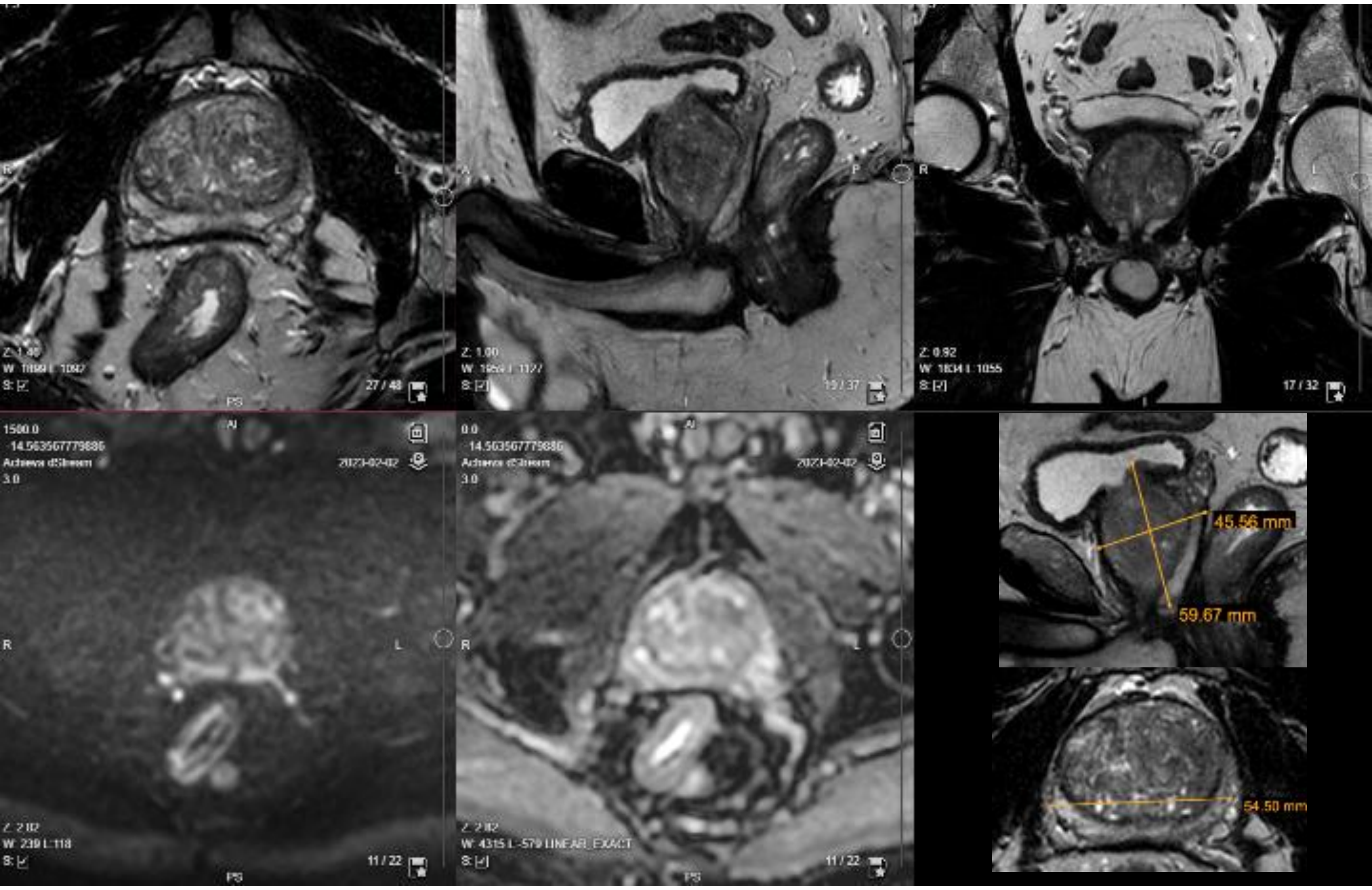

PSA: 12 µg/L. Prostate volume: 77 ml. PSA density: 0.16 µg/L/ml.
Scanner: Philips Achieva dStream 3T.

DESCRIPTION: Hyperplasia with multiple nodules in the transition zone. Striated changes in the peripheral zone without significant diffusion restriction. Summary assessment PI-RADS 2.

CLINICAL MANAGEMENT: Systematic biopsy due to PSA density slightly above the threshold value. Benign histology.

DISCUSSION: Most of the changes seen on the morphological images are benign, since there is no diffusion restriction within those areas.

DIFFICULTY LEVEL: Easy

EQUALIS – PROSTATE MRI

Case 2. Expected response: PI-RADS 3, TZ

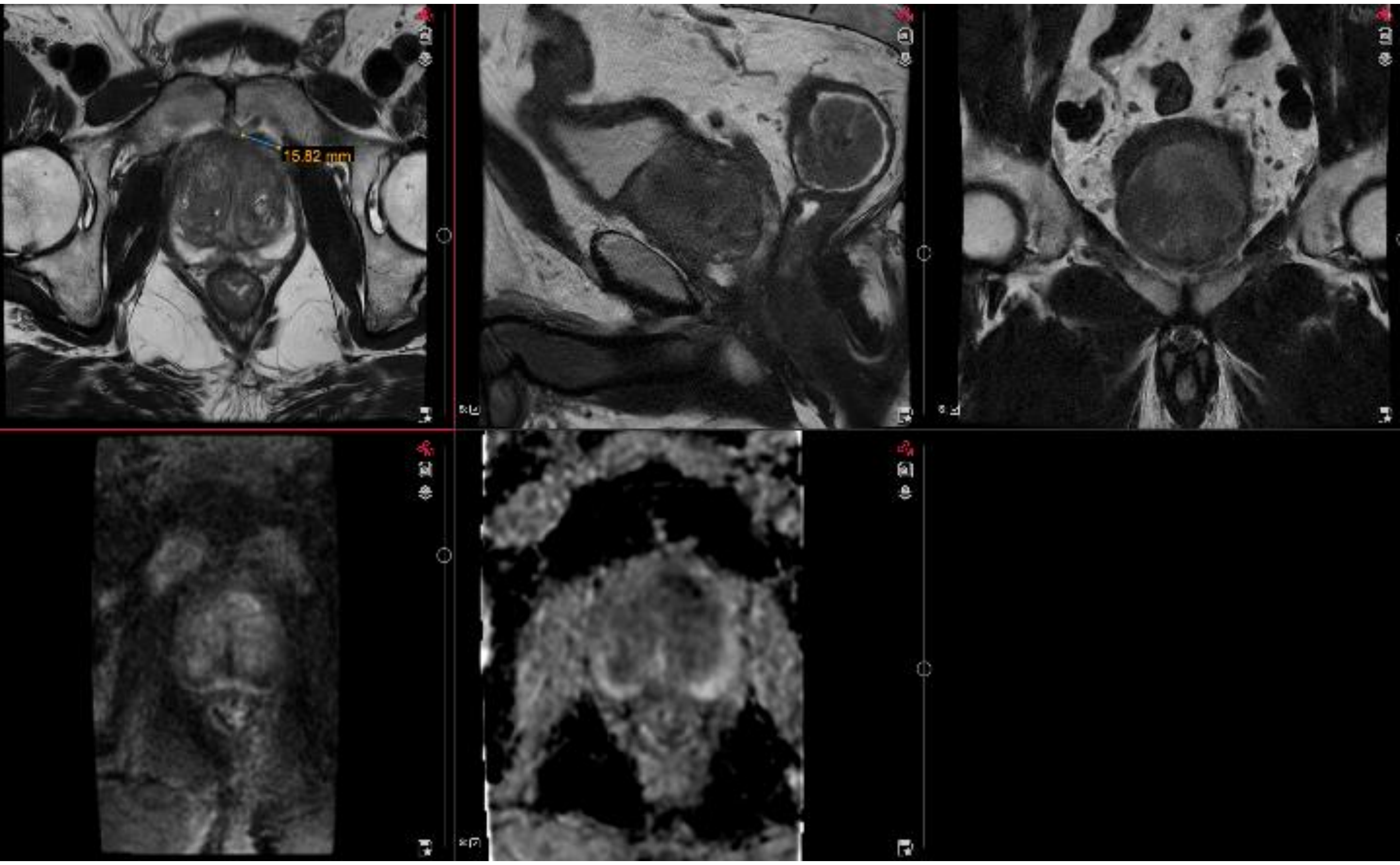

PSA: 8 µg/L. Prostate volume: 143 ml. PSA density: 0.06 µg/L/ml.
Scanner: GE, Signa Premier, 3T.

DESCRIPTION: Ventrally in the transition zone on the left side, sector 3Bv, there is a 16 mm area between nodules with restricted diffusion, PI-RADS 3.

SIGNIFICANT INCIDENTAL FINDING: Larger stalked tumor in the rectum. Clear diffusion restriction within the tumor.

CLINICAL MANAGEMENT: According to the recommendation in the national guidelines, no biopsy was performed (PI-RADS 3 with low PSAD). Follow-up PSA control. Referred to the surgeon for further investigation of rectal polyp.

DISCUSSION: Suspected tumor changes in the rectum are a relatively common incidental finding on MR prostate.

DIFFICULTY LEVEL: Medium

EQUALIS – PROSTATE MRI

Case 2. Expected response: PI-RADS 3, TZ

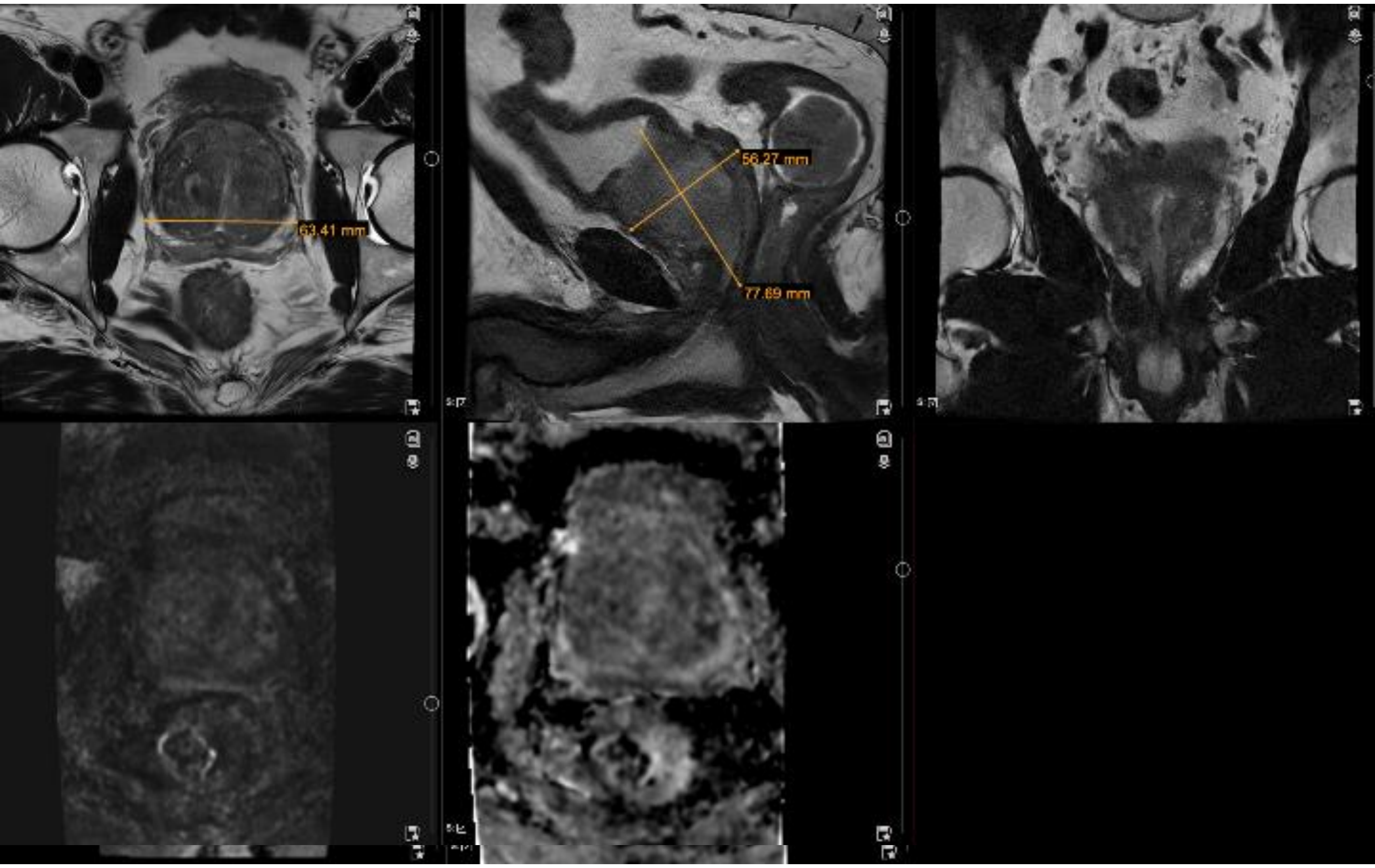

Volume measurement example.

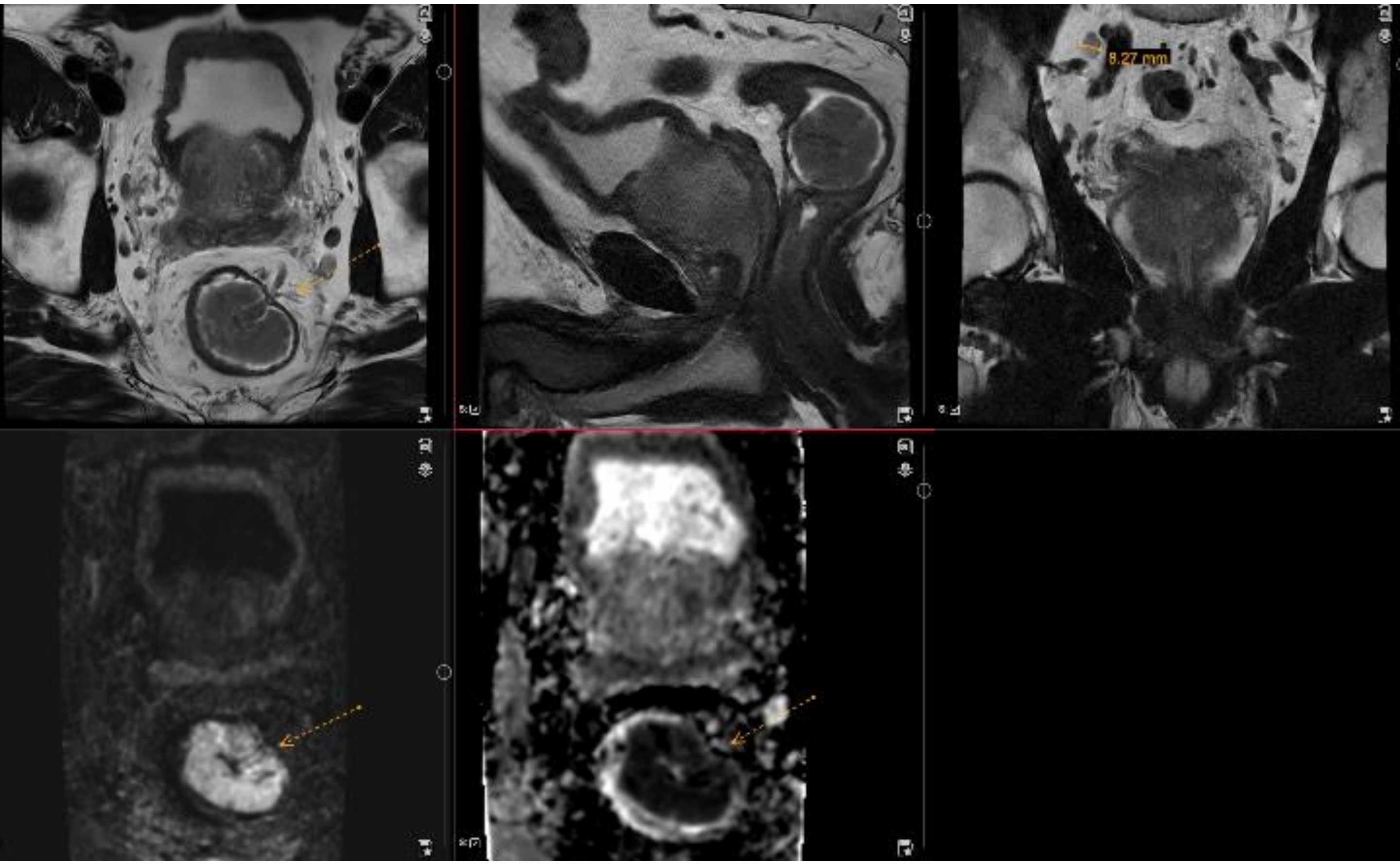

Rectal polyp

EQUALIS – PROSTATE MRI

Case 3. Expected response: PI-RADS 5, PZ

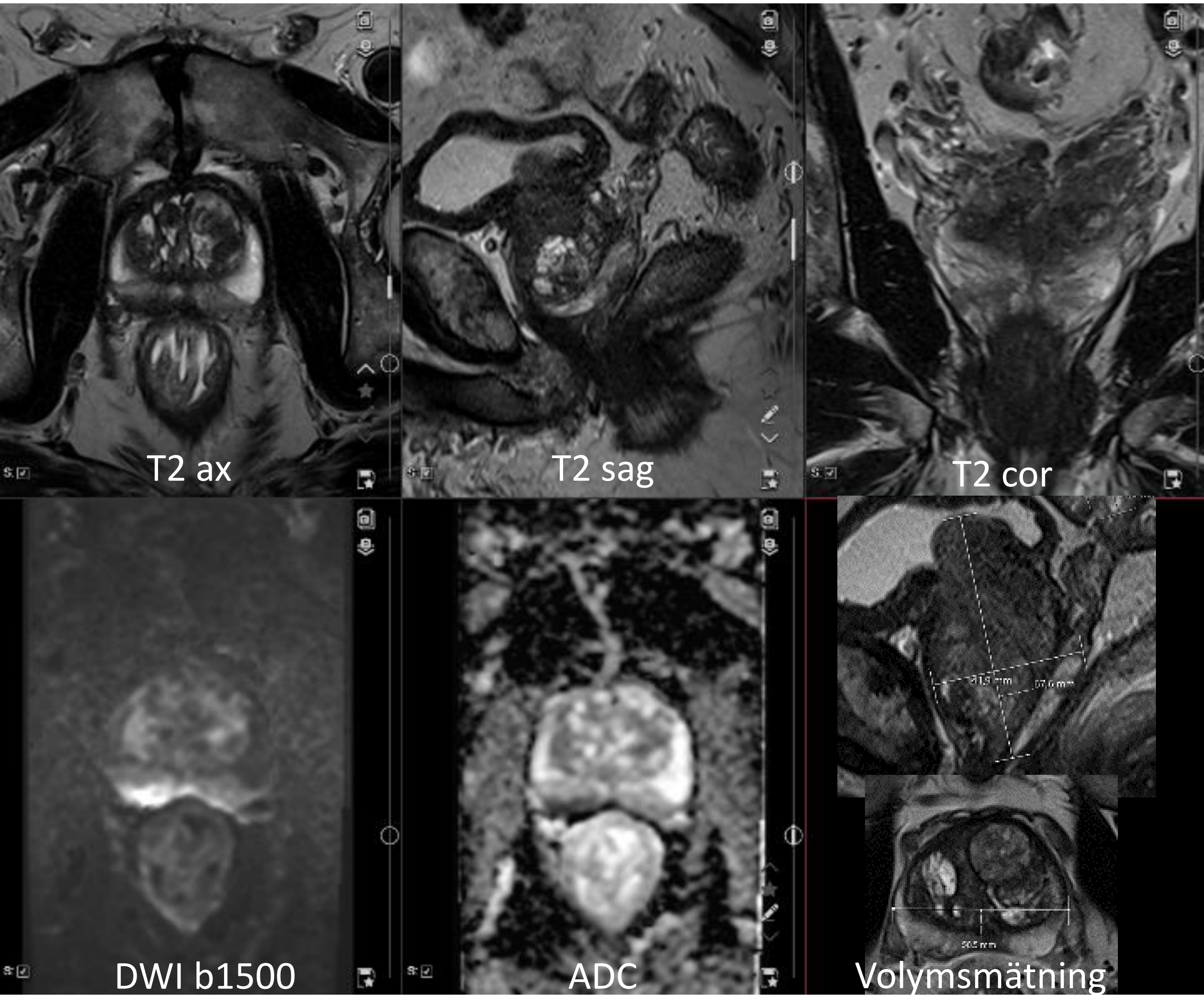

PSA: 7.1 µg/L. Prostate volume: 87 ml with PSA density: 0.08 µg/L/ml.
Scanner: Siemens, Magnetom Vida 3T.

DESCRIPTION: Focal lesion in the peripheral zone dorsally on the right side in the midportion/apex (1BCd) with focal and pronounced diffusion restriction (ADC value approx. 600), size on ADC 17 mm, corresponds to T2 of focal low-signal lesion with extension along the capsule, PI-RADS 5. Interrupted and irregular capsule on T2 >12 mm, no convincing extraprostatic diffusion restriction and assessed EPE 4/5. The lesion does not touch the seminal vesicles, SVI 1/5.

CLINICAL MANAGEMENT:
MR-guided biopsies against PI-RADS 5 with Gleason grade 3+4. RARP (tumor map attached) pT3A with largest tumor focus (19 x 8 mm) correlating to PI-RADS 5, Gleason 3+4 (10% grade 4), minimal EPE dorsolaterally right. Cancer-positive resection margins in apex right (Gleason 3+3). No SVI.

DISCUSSION: Sagittal image planes show larger extension craniocaudally which is a common growth pattern where there is a risk of underestimating tumor size.
DIFFICULTY LEVEL: Medium

EQUALIS – PROSTATE MRI

Case 3. Histology from whole-mount prostate specimen

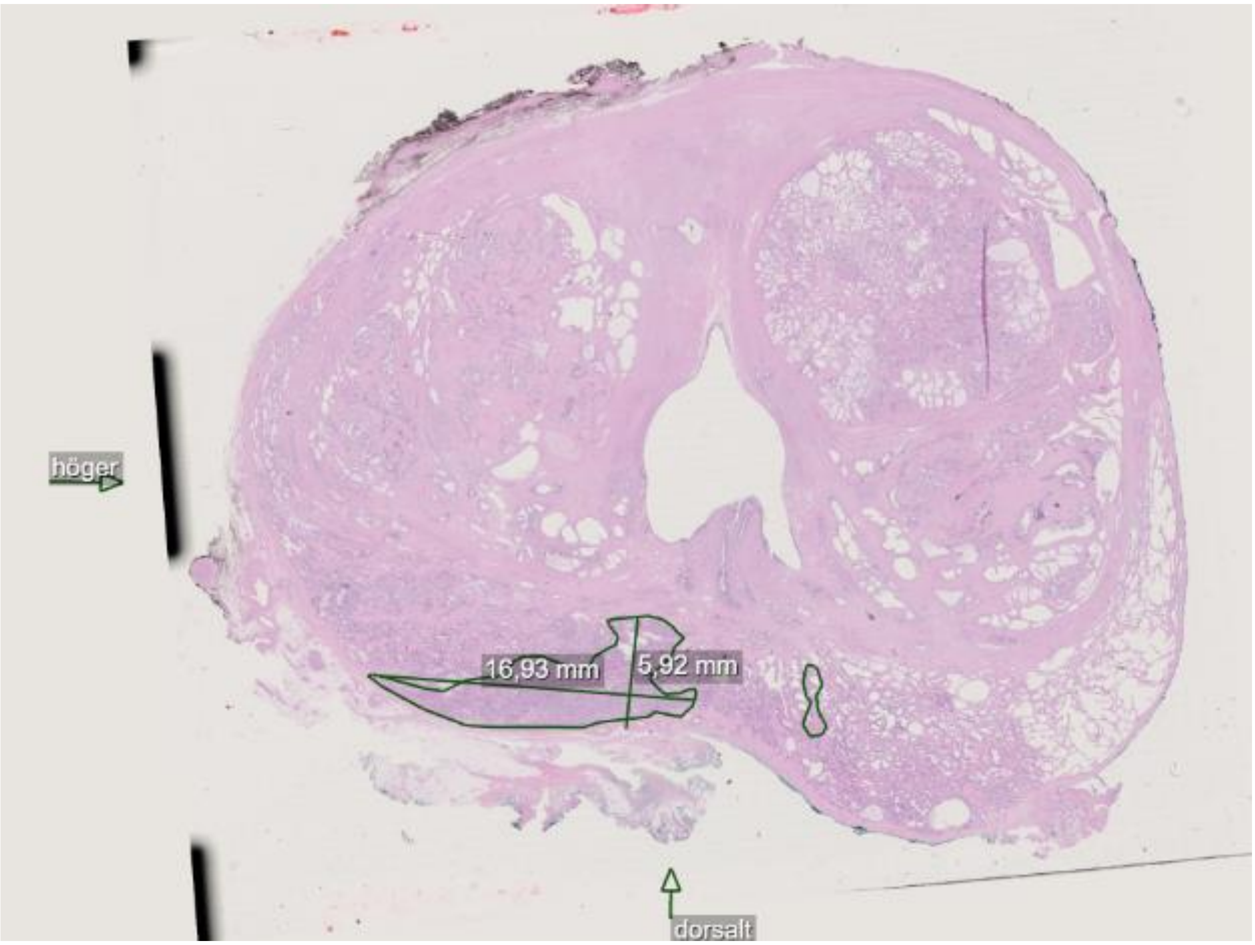

Histological tumor map, corresponding to the PI-RADS 5 lesion. Gleason 3+4.

EQUALIS – PROSTATE MRI

Case 4. Expected response: PI-RADS 2

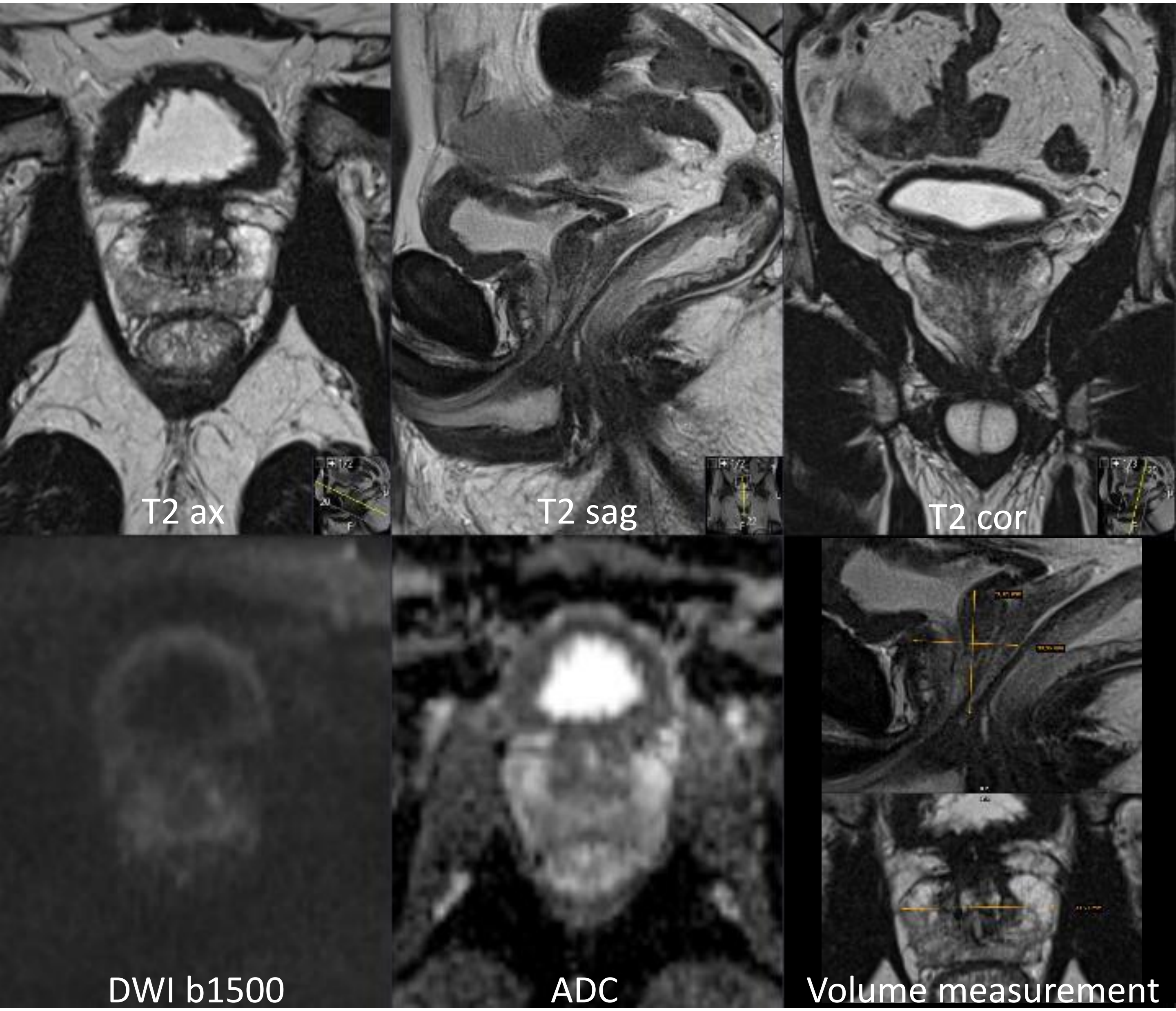

PSA: 3.40 µg/L. Prostate volume: 22 ml with PSA density: 0.15 µg/L/ml.
Scanner: Siemens, Magnetom Aera, 1.5T.

DESCRIPTION:

In the peripheral zone, symmetrically low-signal areas with discrete diffusion restriction are seen in the dorsal and dorsolateral extent, with clear demarcation against normal peripheral zone. No focal abnormal areas with significant diffusion restriction. Transition zone without suspected malignant changes.

MANAGEMENT:

Histology from systematic biopsies, which were taken due to PSA density at the threshold value, showed in 1/12 atypical cells, not enough for cancer diagnosis.

DISCUSSION:

The appearance in the peripheral zone may represent previous prostatitis or systematic biopsy.

DIFFICULTY LEVEL: Medium

EQUALIS – PROSTATE MRI

Case 4. Cont. Volume measurement

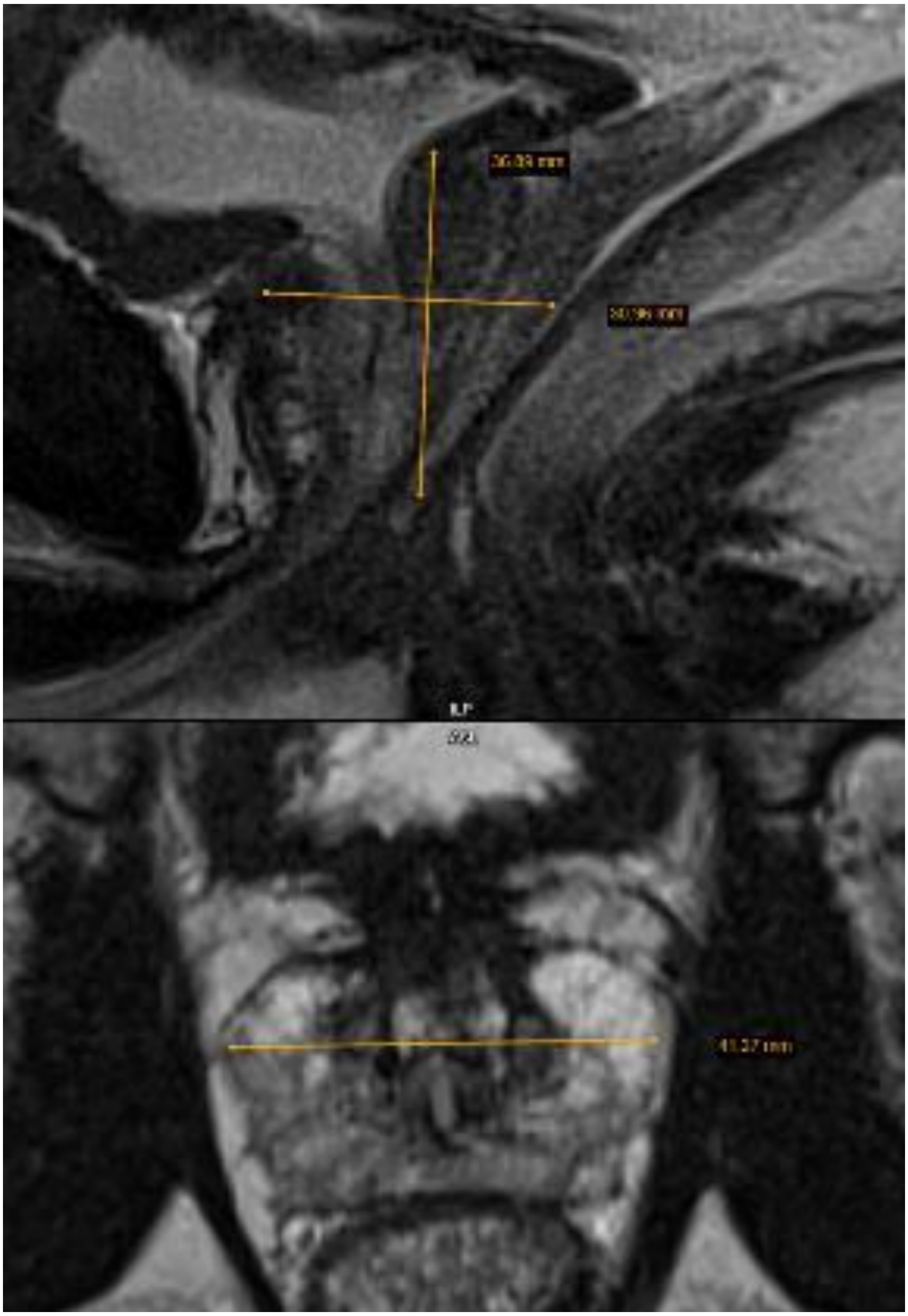

PSA: 3.40 µg/L. Prostate volume: 22 cc with PSA density 0.15 µg/L/cc.
When measuring the volume, it is important to include the entire prostate in the different measurements. On the sagittal image, the height and depth are measured perpendicular to each other in the midline where the urethra is visualized. The width is measured where the prostate has its largest width.
The volume is calculated according to the ellipsoid formula B x D x H x 0.52.
It is important that the prostate volume is correct as it forms the basis for the PSA density calculation. In this case, a volume of 24 cc is obtained with the specified dimensions above, which gives a PSA density of 0.14 µg/L/cc and since the current limit for biopsies is 0.15 µg/L/cc, one could have refrained from biopsies.

EQUALIS – PROSTATE MRI

Case 5. Expected response: PI-RADS 3 - PZ

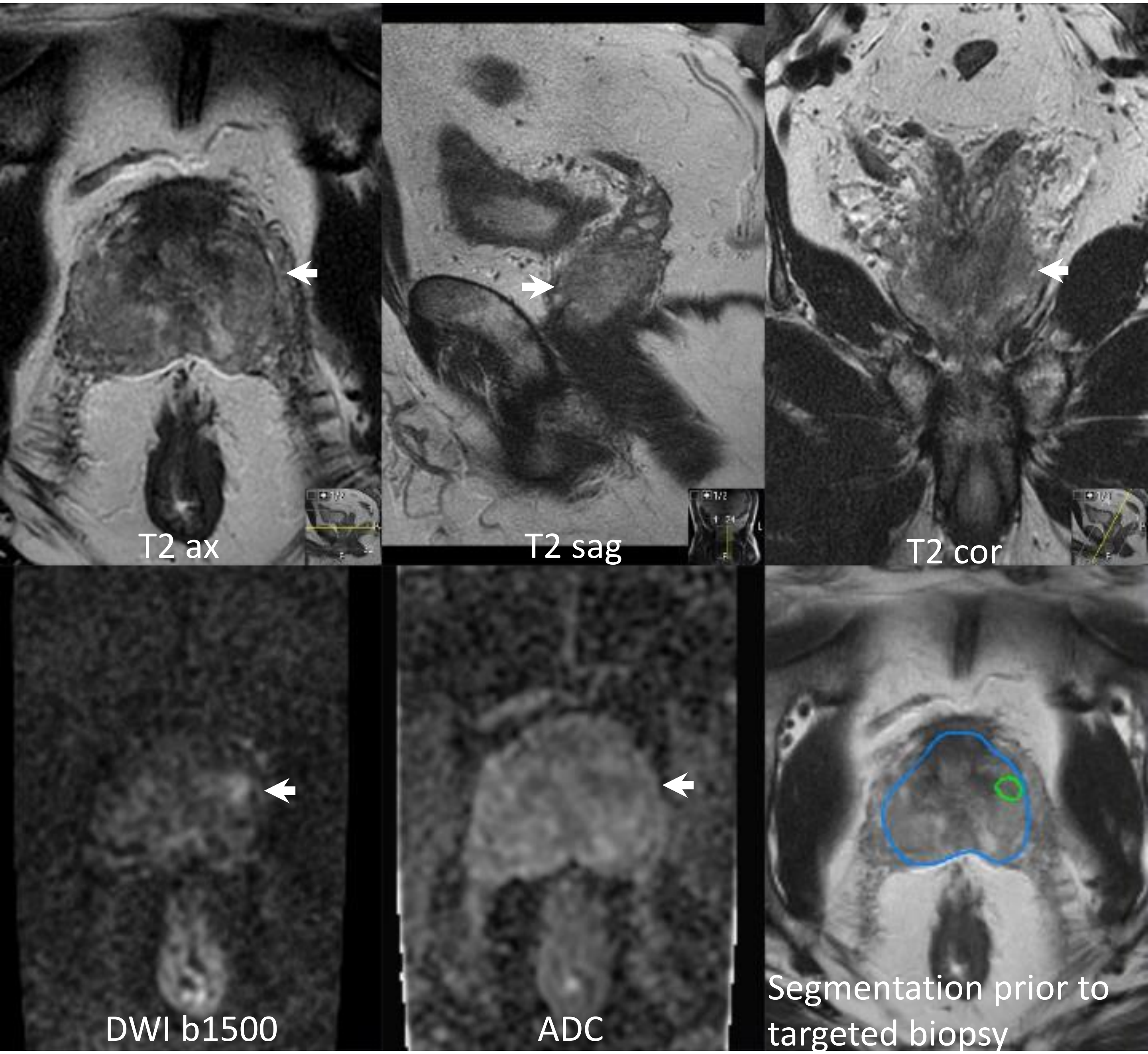

PSA: 2.6 µg/L. Prostate volume: 34 ml. PSA density: 0.08 µg/L/ml.
Scanner: GE, Signa Architect, 3T.

DESCRIPTION: Peripheral zone generally low-signal on T2-weighted images. Ventrolaterally in the base on the left side, an irregularly delimited area (arrows) with significant diffusion restriction, which is barely visible on T2-weighted images. Transition zone without suspected malignant changes.

EQUALIS – PROSTATE MRI

Case 5. Cont.

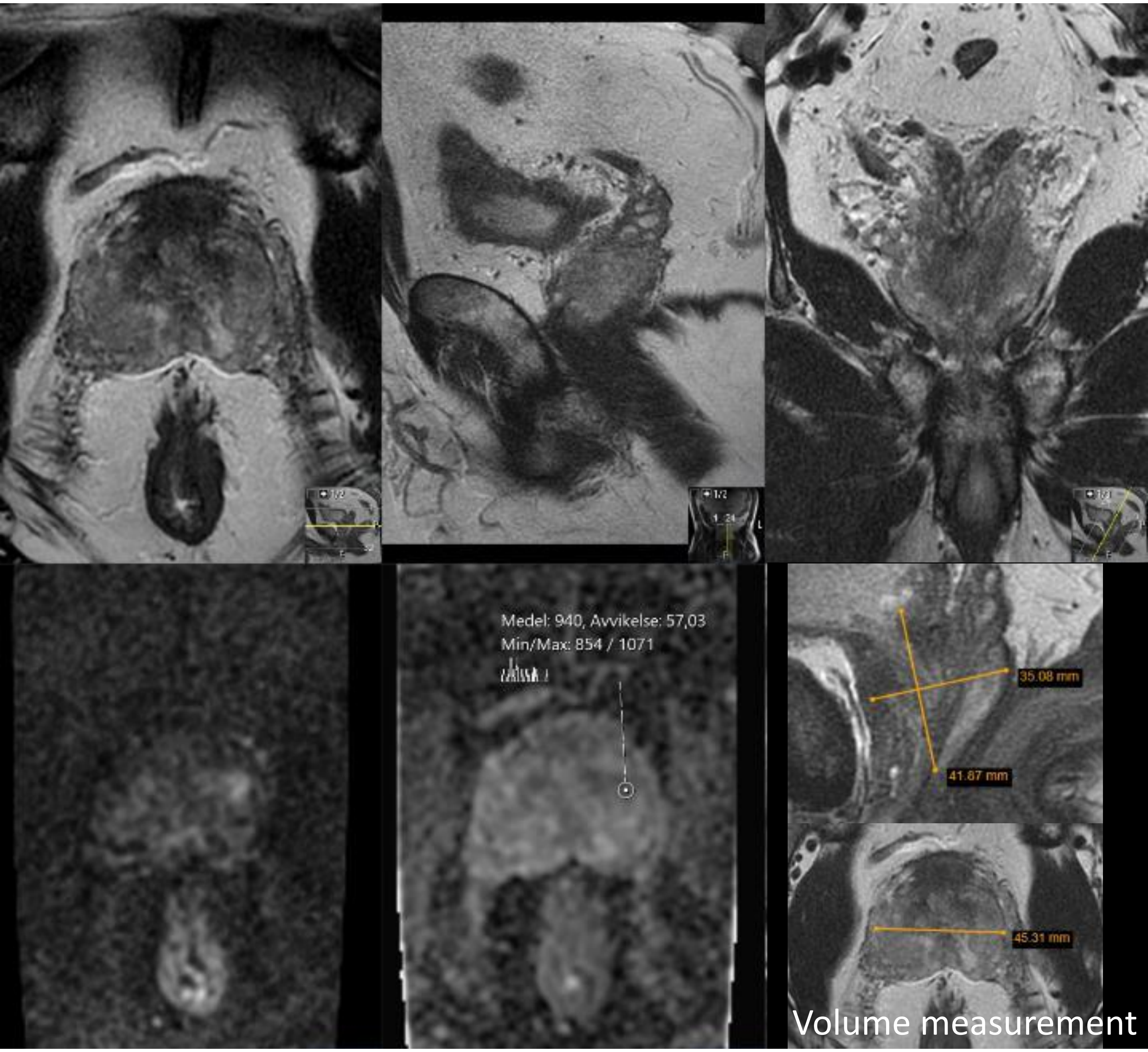

PSA: 2.6 µg/L. Prostate volume: 34 ml. PSA density: 0.08 µg/L/ml.

DESCRIPTION: The area ventrolaterally in the base on the left side has significant diffusion restriction (see measurement on the ADC map).

MANAGEMENT:

Histology from MR-guided biopsies showed in 2/4 Gleason 3+4 with perineural infiltration and mucinous component.

Histology from the postoperative prostate specimen showed acinar adenocarcinoma with mucinous component, Gleason 3+4, pT2, negative resection margins.

DISCUSSION: DWI/ADC dominant sequence in PZ, where the change is difficult to discern on T2w. It is not uncommon in younger men with generally low-signalling PZ, but then without diffusion restriction.

DIFFICULTY LEVEL: Difficult

EQUALIS – PROSTATE MRI

Case 6. Expected response: PI-RADS 4, PZ

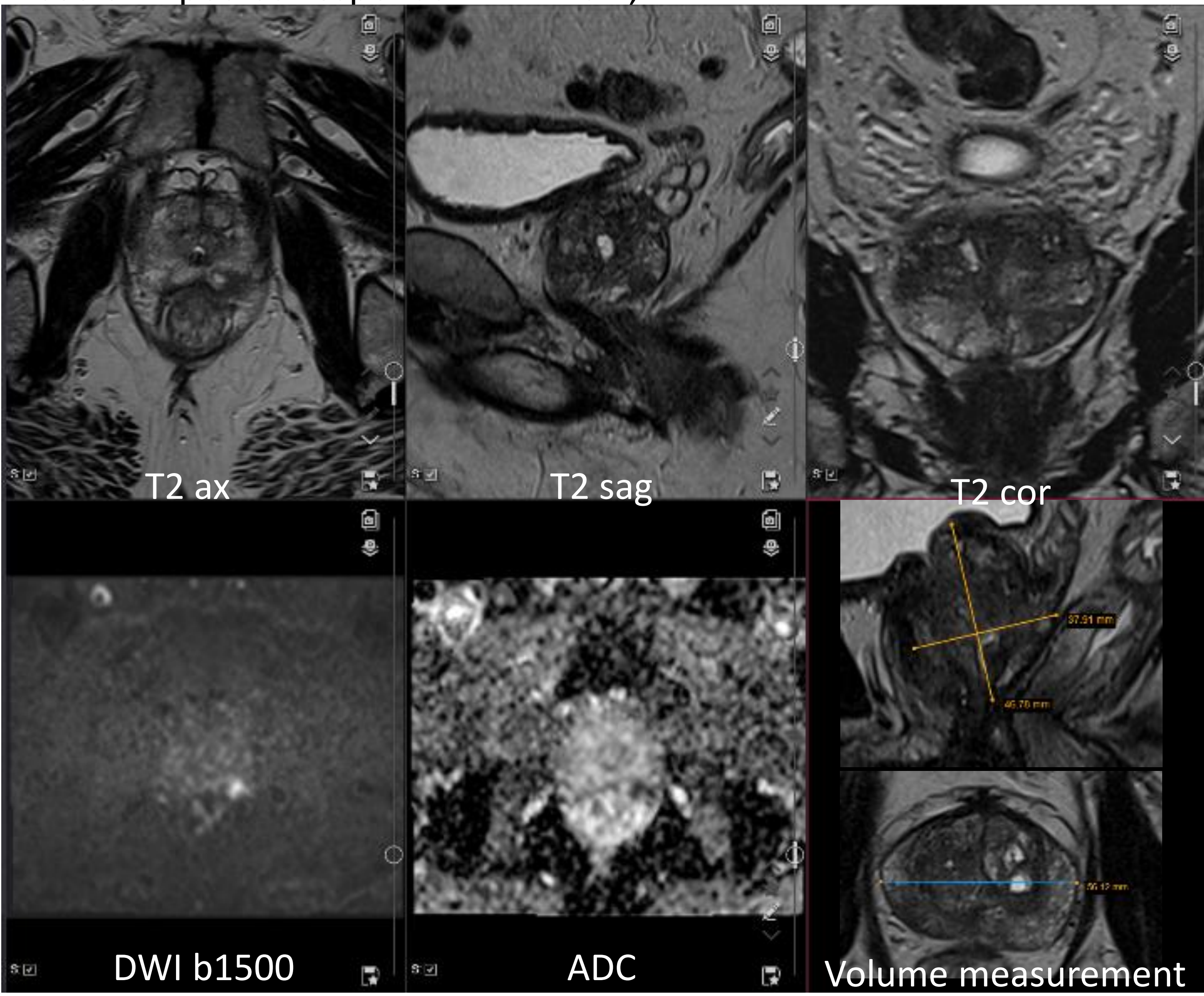

PSA: 5.6 µg/L. Prostate volume: 52 ml with PSA density: 0.11 µg/L/ml.
Scanner: Siemens Magnetom Aera, 1,5T.
DESCRIPTION: In the peripheral zone dorsally on the left side in the apex, sector 4Cd, a focal lesion with pronounced diffusion restriction (ADC value 600) and size 13 mm on ADC, PI-RADS 4, is seen. On T2 low-signal diffusely delimited lesion with wide attachment to the capsule and interrupted capsule line but no measurable radial growth outside the capsule, interpreted EPE 4/5. The lesion is not located near the seminal vesicles, SVI 1/5. In addition, there is a focal lesion in the peripheral zone right base, sector 1Ad with some diffusion restriction (ADC 950), size 8 mm, PI-RADS 3.
MANAGEMENT: MR-guided biopsies against PI-RADS 4 with Gleason grade 3+4 and 3 right-sided non-directed biopsies with Gleason grade 3+3 in 3Bd.
RARP (tumor map attached) pT3A with largest tumor focus correlating to PI-RADS 4 Gleason 3+4 (40% grade 4), cribriform growth grade 4 and EPE dorsolaterally left, cancer-free resection margins. On tumor map tumor focus correlating to PI-RADS 3 with Gleason grade 3+3.
DISCUSSION: Sagittal T2 confirms that the focal diffusion restriction corresponds to an intraprostatic lesion in 4Cd. EPE assessment in the base and apex is difficult on axial images due to partial volume effects that make the capsule blurred. In this case, EPE is best assessed on sagittal series.
PI-RADS 3 lesion in right base was overlooked at original review.
DIFFICULTY LEVEL: Difficult

Case 6. Cont.

EQUALIS – PROSTATE MRI

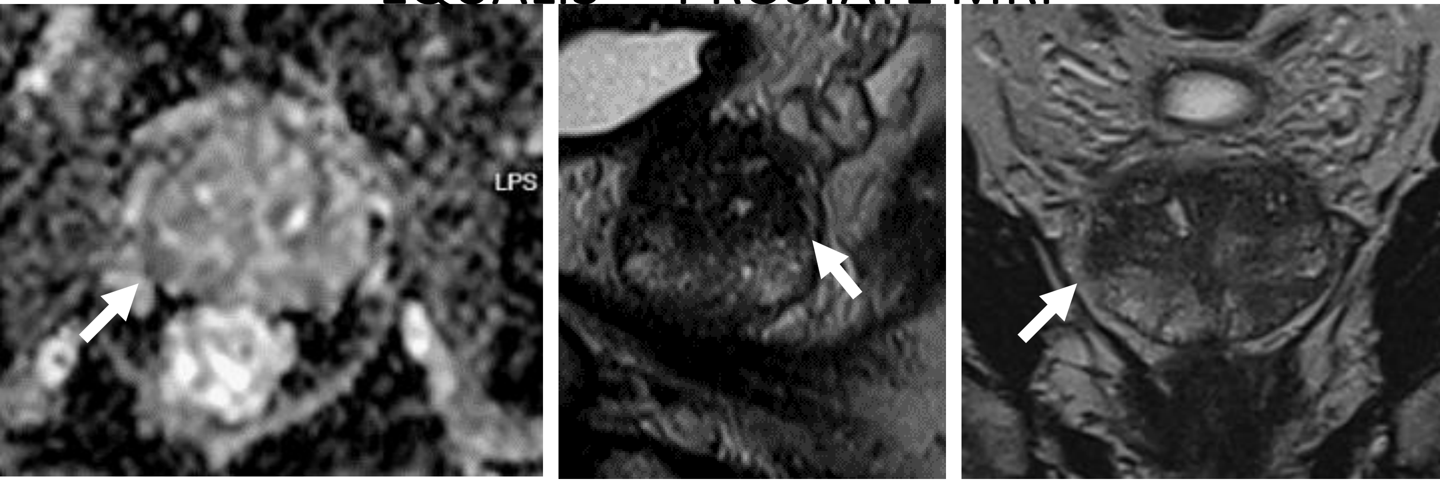

PI-RADS 3, PZ, 1Ad

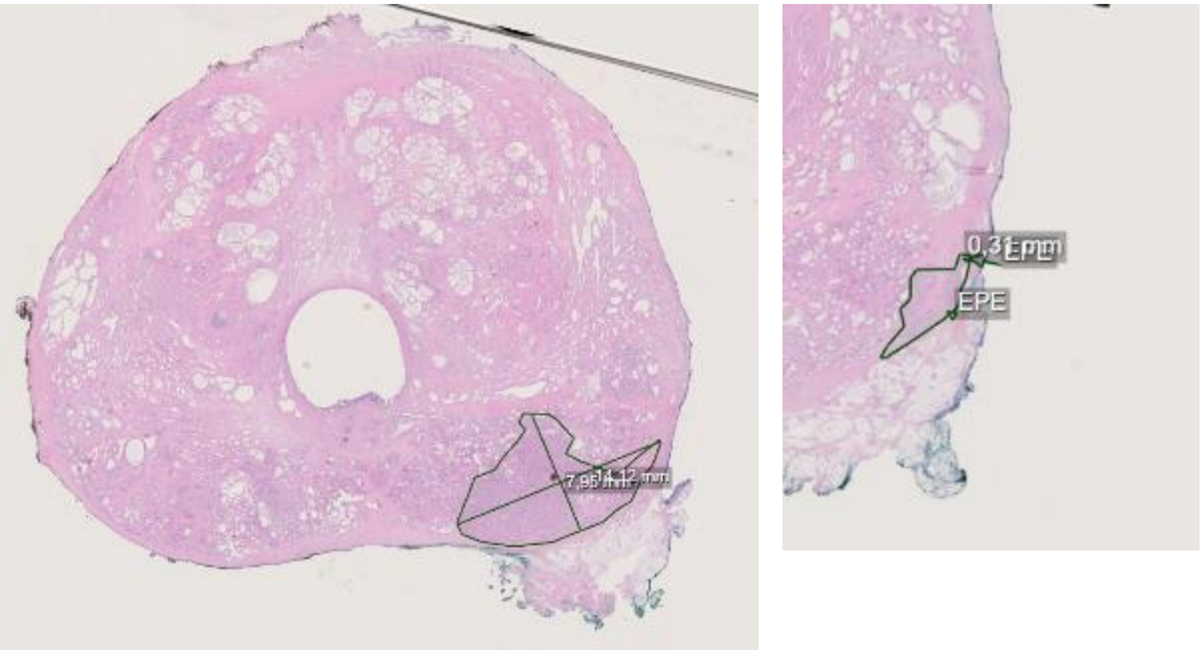

Histological tumor map, corresponding to the PI-RADS 4 lesion. Gleason 3+4 with extra-prostatic extension.

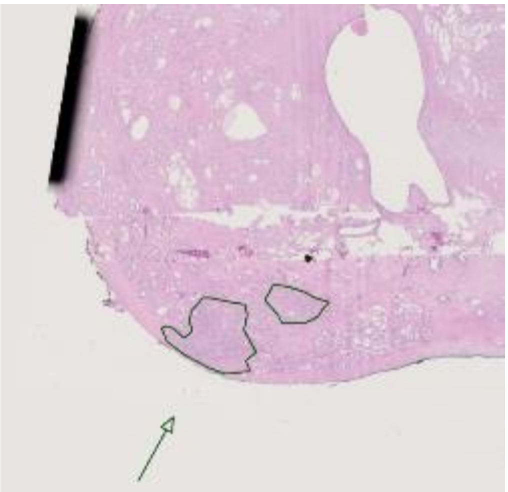

Histological tumor map, corresponding to the PI-RADS 3 lesion. Gleason 3+3.

EQUALIS – PROSTATE MRI

Case 7. Expected response: PI-RADS 2

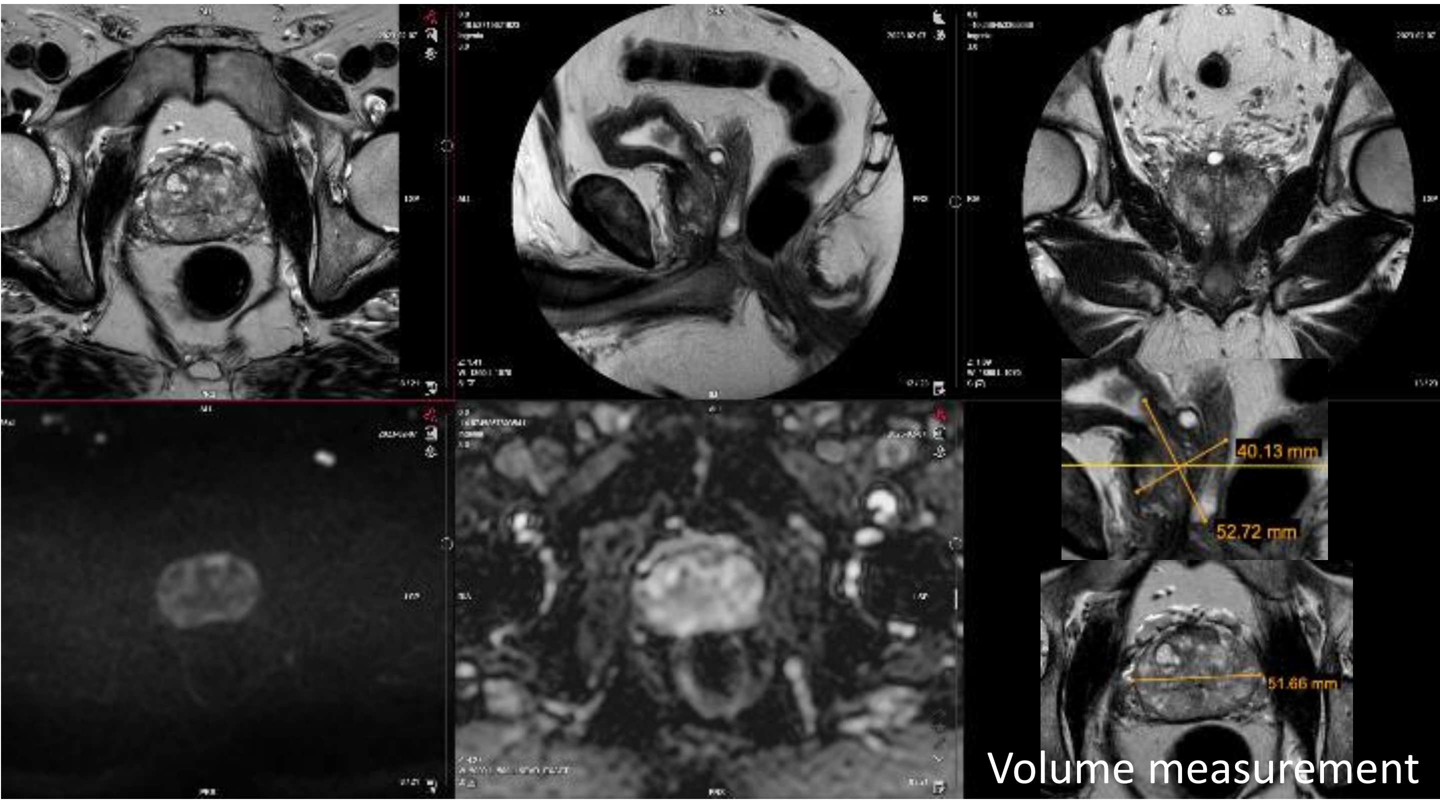

PSA: 4 µg/L. Prostate volume: 57 ml. PSA density: 0.07 µg/L/ml.
Scanner: Philips, Ingenia, 3T.

DESCRIPTION: Hyperplasia of the transition zone with multiple nodules. Striated changes in the peripheral zone without significant diffusion restriction. Summary assessment PI-RADS 2.

SIGNIFICANT INCIDENTAL FINDING: There is a ca 2 cm large diffusion-restricted tumor change in the urinary bladder dorsolaterally left.

CLINICAL MANAGEMENT: No biopsy (PI-RADS 2, low PSAD). Cystoscopy.

DISCUSSION: Multiple nodules in the transition zone including scattered areas with mild diffusion restriction "organized chaos". No changes that grow over the nodule boundaries or that exhibit strongly deviating diffusion.

DIFFICULTY LEVEL: Medium

EQUALIS – PROSTATE MRI

Case 7. Expected response: PI-RADS 2

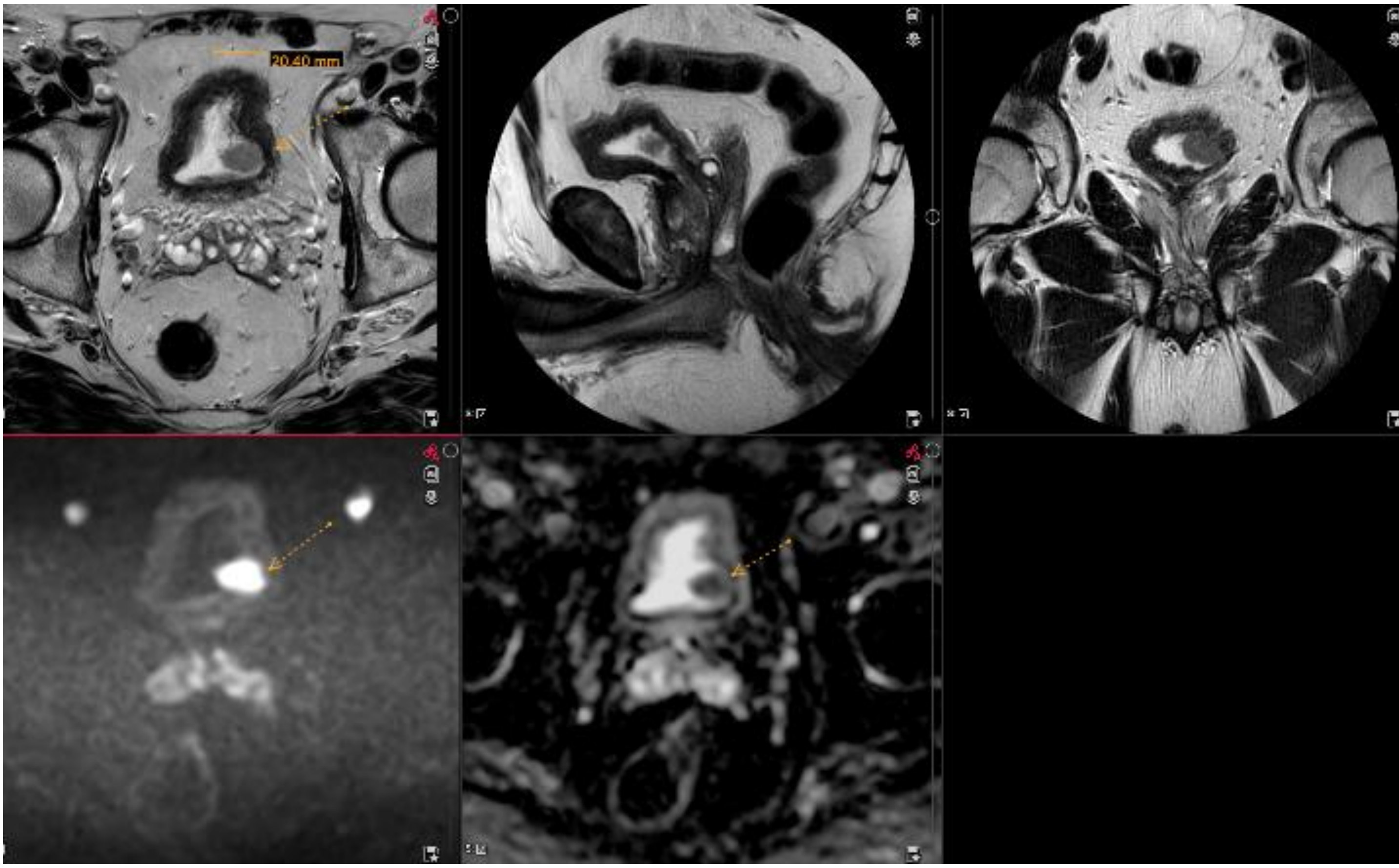

SIGNIFICANT INCIDENTAL FINDING: There is a ca 2 cm large diffusion-restricted lesion in the urinary bladder dorsolaterally left. After TUR-B histological diagnosis non-muscle-invasive bladder cancer.

EQUALIS – PROSTATE MRI

Case 8. Expected response: PI-RADS 5, TZ

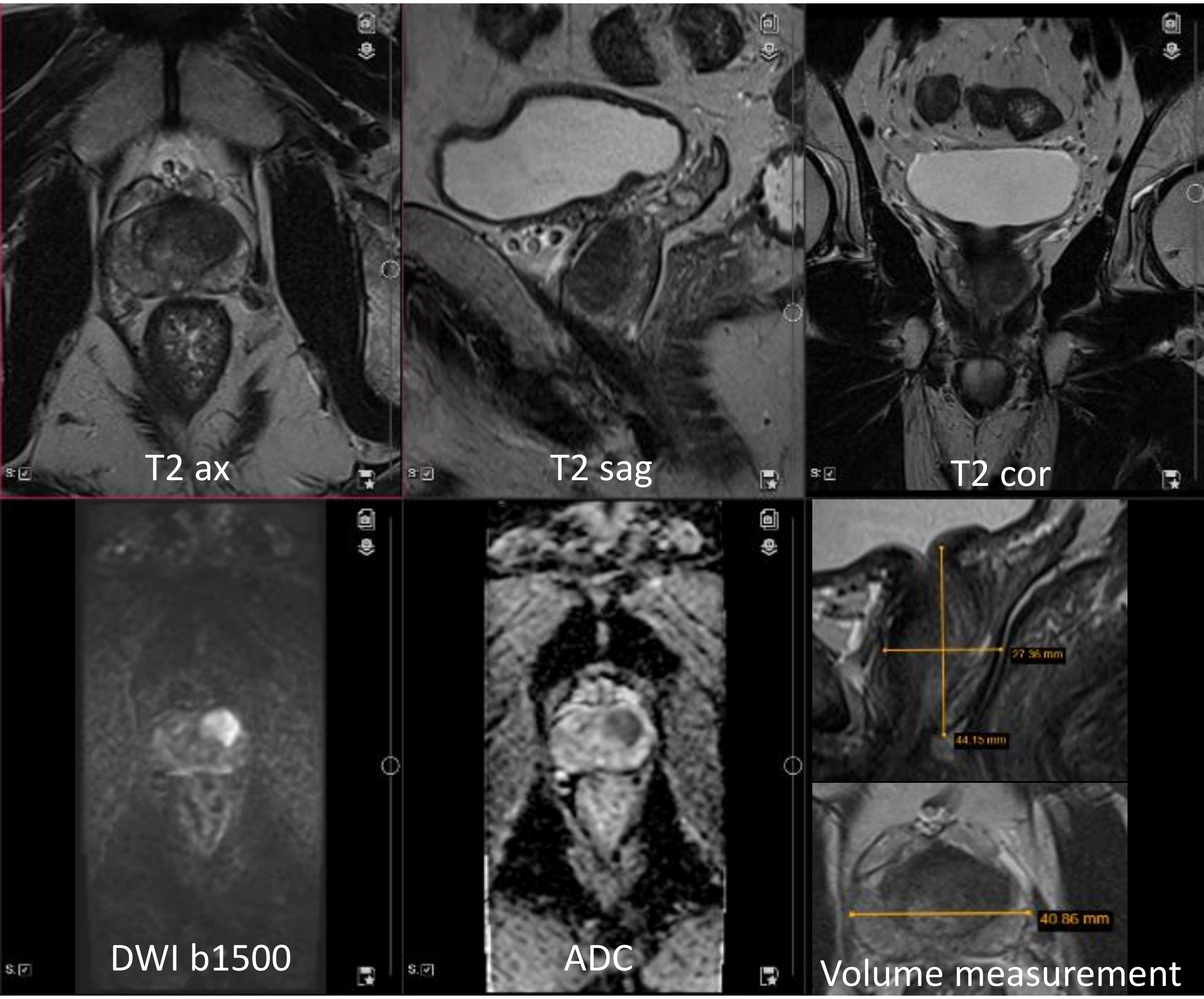

PSA: 3.9 µg/L. Prostate volume: 25 ml with PSA density: 16 µg/L/ml.
Scanner: Siemens, Magnetom Vida, 3T.

DESCRIPTION: Originating from the transition zone ventrally on the left side extending from the base down to the apex, a diffuse delimited low-signaling lesion with pronounced diffusion restriction (ADC value approximately 650 (435-784)) is seen on T2w. The lesion measures over 15 mm in size and is classified as PI-RADS 5. Long abutment to the capsule >12 mm without indirect signs, EPE 3/5. No proximity to the seminal vesicles SVI 1/5.

MANAGEMENT: The patient was included in organized prostate cancer testing (OPT) and was handled according to this routine. MR-guided biopsies showed Gleason 3+4. RARP with tumor map pT2 (see attached example) shows Gleason 3+4 (15% grade 4), no extraprostatic extension, no seminal vesicle invasion, cancer-free resection margins.

DISCUSSION: The lesion does not show encapsulation and its shape is not typical for nodule. It may be of value to create a sagittal MPR on ADC to get an idea of the lesion's extent (see attached example).

DIFFICULTY LEVEL: Medium

Case 8. Cont.

EQUALIS – PROSTATE MRI

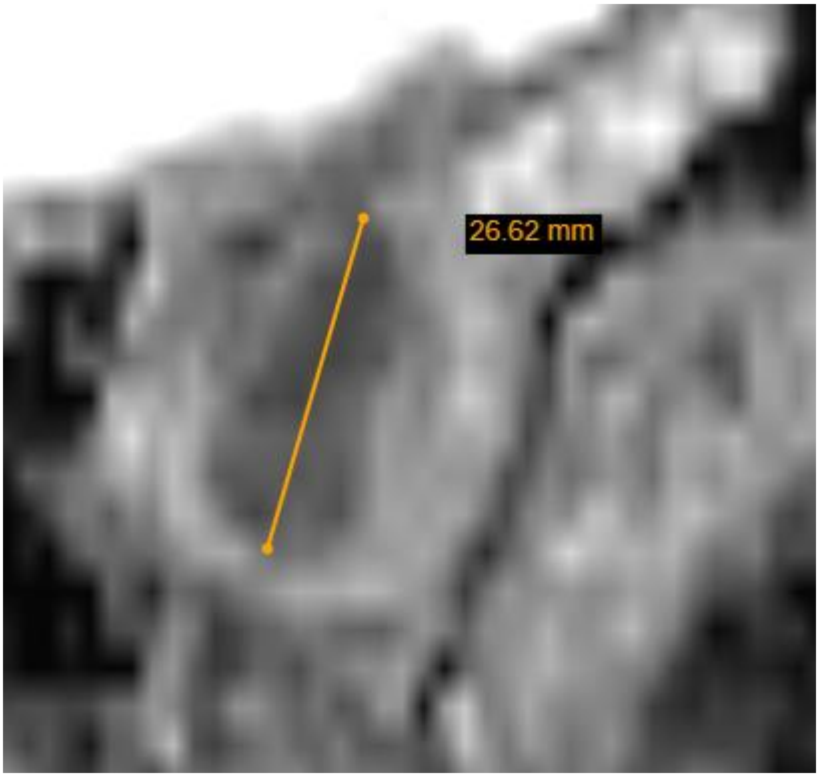

Sagittal reconstruction of the ADC-map reveals a large non-nodular lesion.

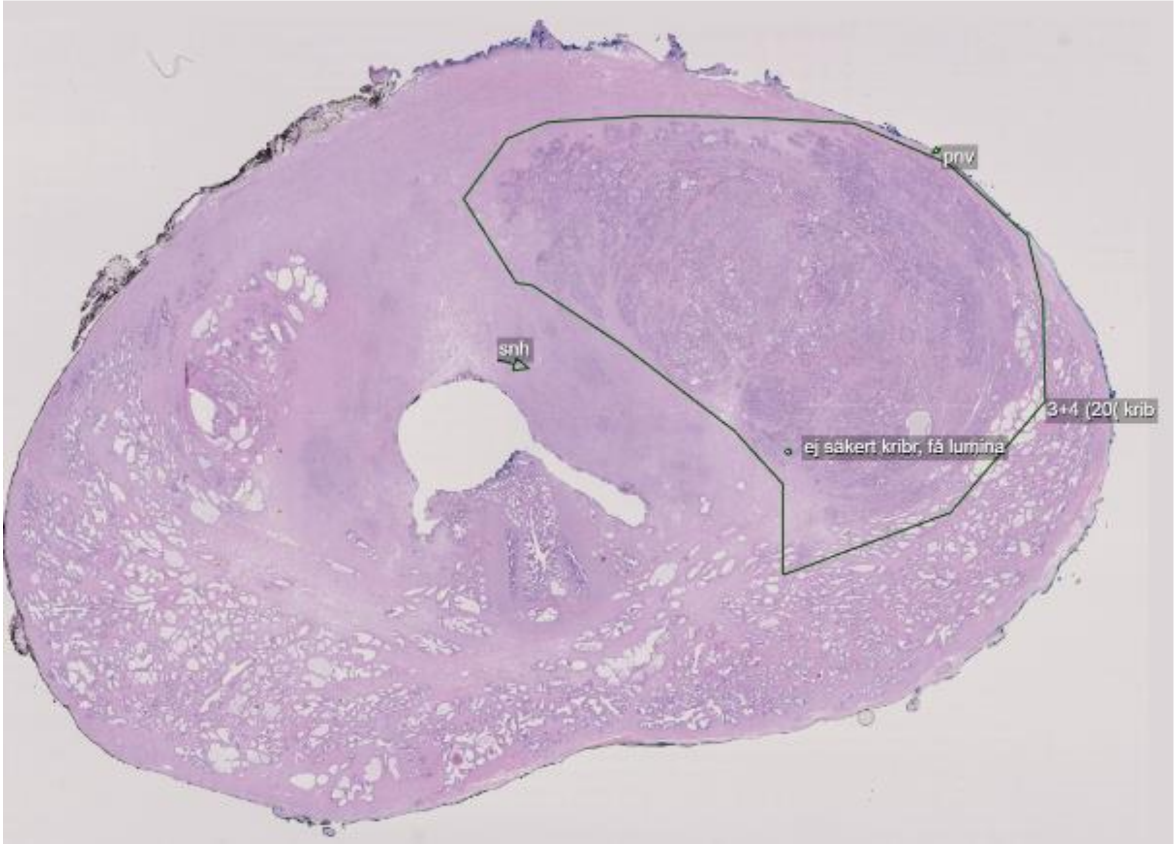

Histology from whole-mount prostate specimen with the lesion corresponding to the PI-RADS 5 lesion on MRI, Gleason 3+4, no extraprostatic extension.

EQUALIS – PROSTATE MRI

Case 9. Expected response: PI-RADS 3 - TZ

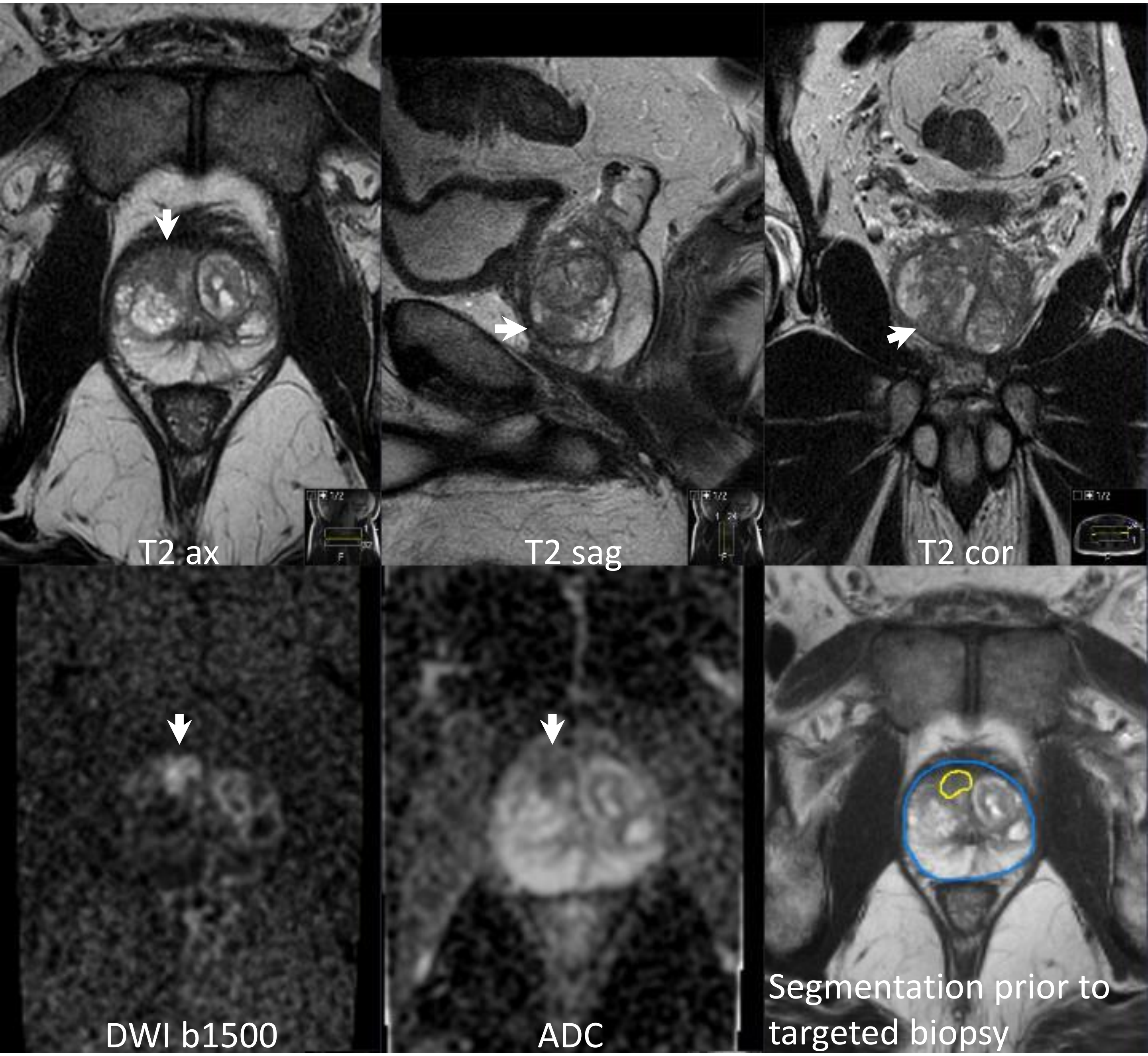

PSA: 4.0 µg/L. Prostate volume: 63 ml. PSA density: 0.06 µg/L/ml.
Scanner: GE, Signa Architect, 3T.

DESCRIPTION: Heterogeneous transition zone with several nodules. Ventral to the right in TZ in the apex there is a low-signalling area on T2-weighted images. The area is assessed as irregular change between nodules (see arrows), with significant diffusion restriction.
The area on T2w is assessed PI-RADS 2 and DWI/ADC is assessed PI-RADS 4, which gives a final score PI-RADS 3.

EQUALIS – PROSTATE MRI

Case 9. Cont.

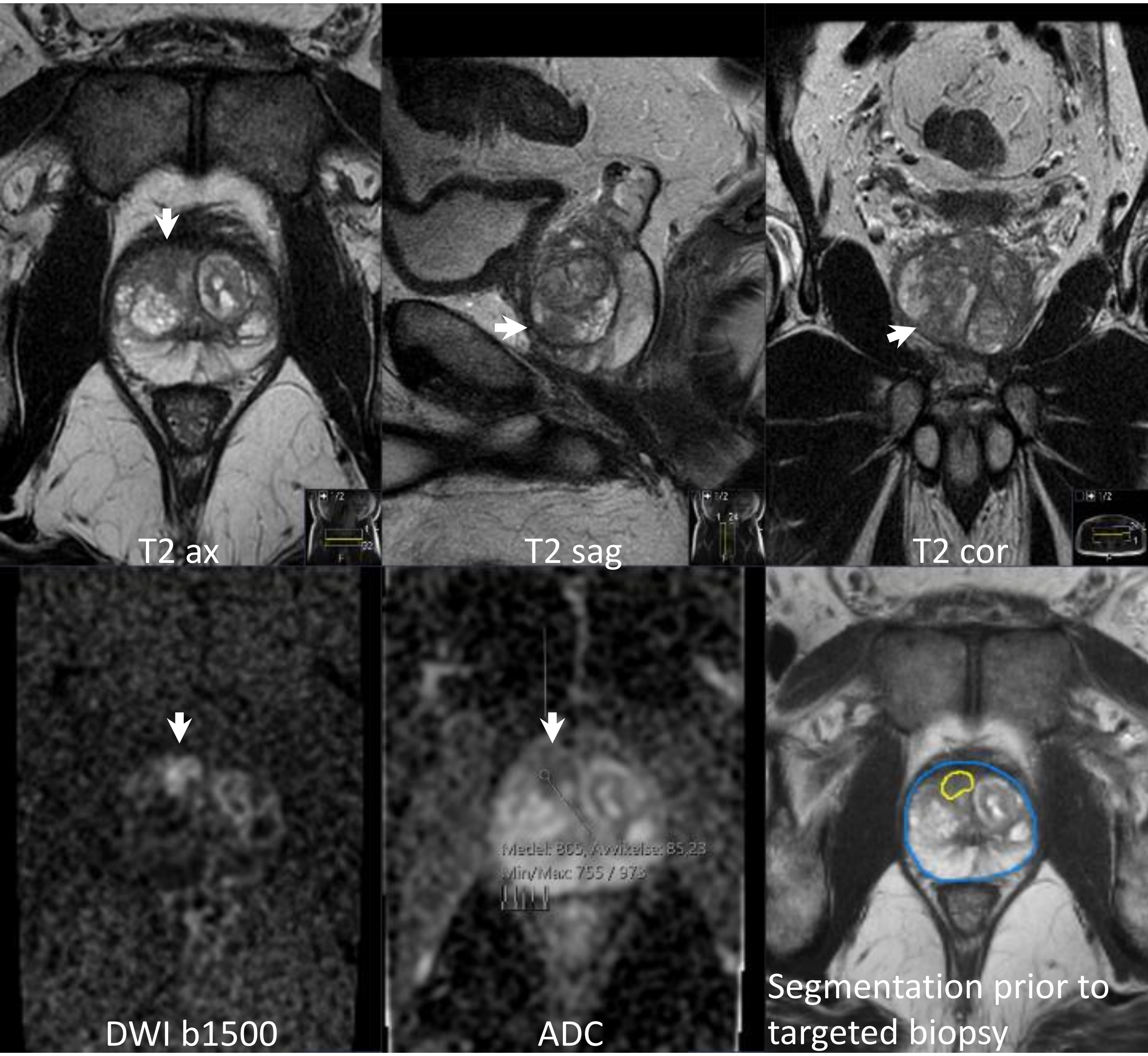

DESCRIPTION:
Low ADC value at measurement.

MANAGEMENT:
MR-guided biopsies against the area ventral to the right in the apex showed no cancer, but inflammation.

DIFFICULTY LEVEL: Medium

EQUALIS – PROSTATE MRI

Case 9. Cont. Follow-up examination 3 years later

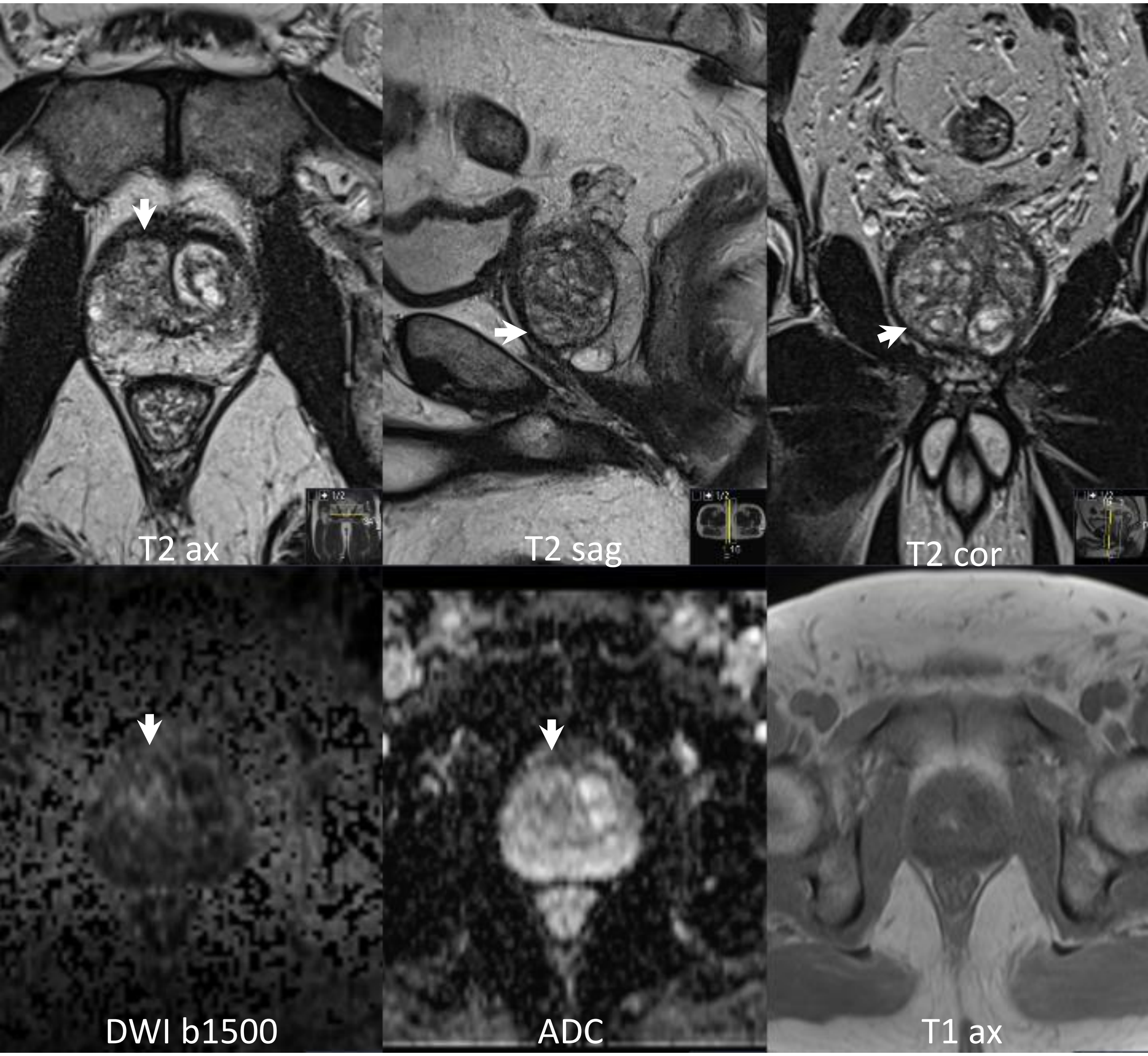

PSA: 6.3 µg/L. Prostate volume: 70 ml. PSA density: 0.09 µg/L/ml.
DESCRIPTION: The low-signal area ventral to the right in the apex has completely regressed. No residual diffusion restriction. No suspected tumor in the prostate.

DISCUSSION: A limitation of prostate MRI is that we cannot distinguish between tumor and widespread inflammation. In benign biopsies, it is desirable that the pathologist describes whether there is inflammation, as a receipt that we have not made a misjudgment.

EQUALIS – PROSTATE MRI

Case 10. Expected response: PI-RADS 3, PZ

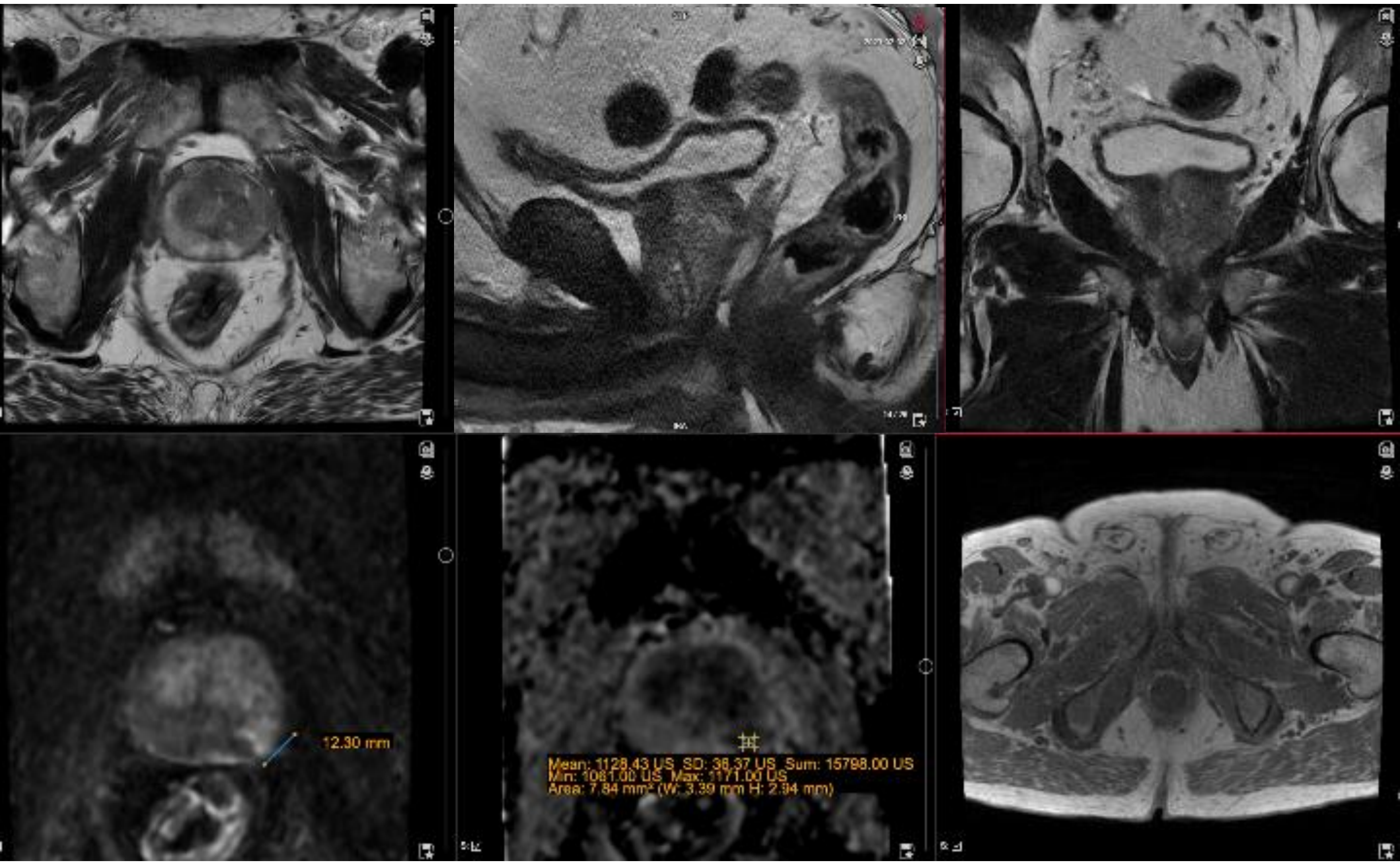

PSA: 9 µg/L. Prostate volume: 55 ml. PSA density: 0.16 µg/L/ml.
Scanner: GE, Signa Premier, 3T.

DESCRIPTION: On T2w diffuse reduced signal in the peripheral zone dorsolaterally on the left side, sector 4Bd, with a 12 mm large area with slightly restricted diffusion (ADC approx. 1000-1100 mm/s2), PI-RADS 3.

MANAGEMENT: PI-RADS 3 with PSA density slightly above the threshold value. Benign histology in directed and systematic biopsy.

DISCUSSION: The change is streaky/sparse on T2 and has slightly restricted diffusion, PI-RADS 3. Only visual assessment of DWI/ADC can lead to overgrading.

DIFFICULTY LEVEL: Medium

EQUALIS – PROSTATE MRI

Case 10. Cont.

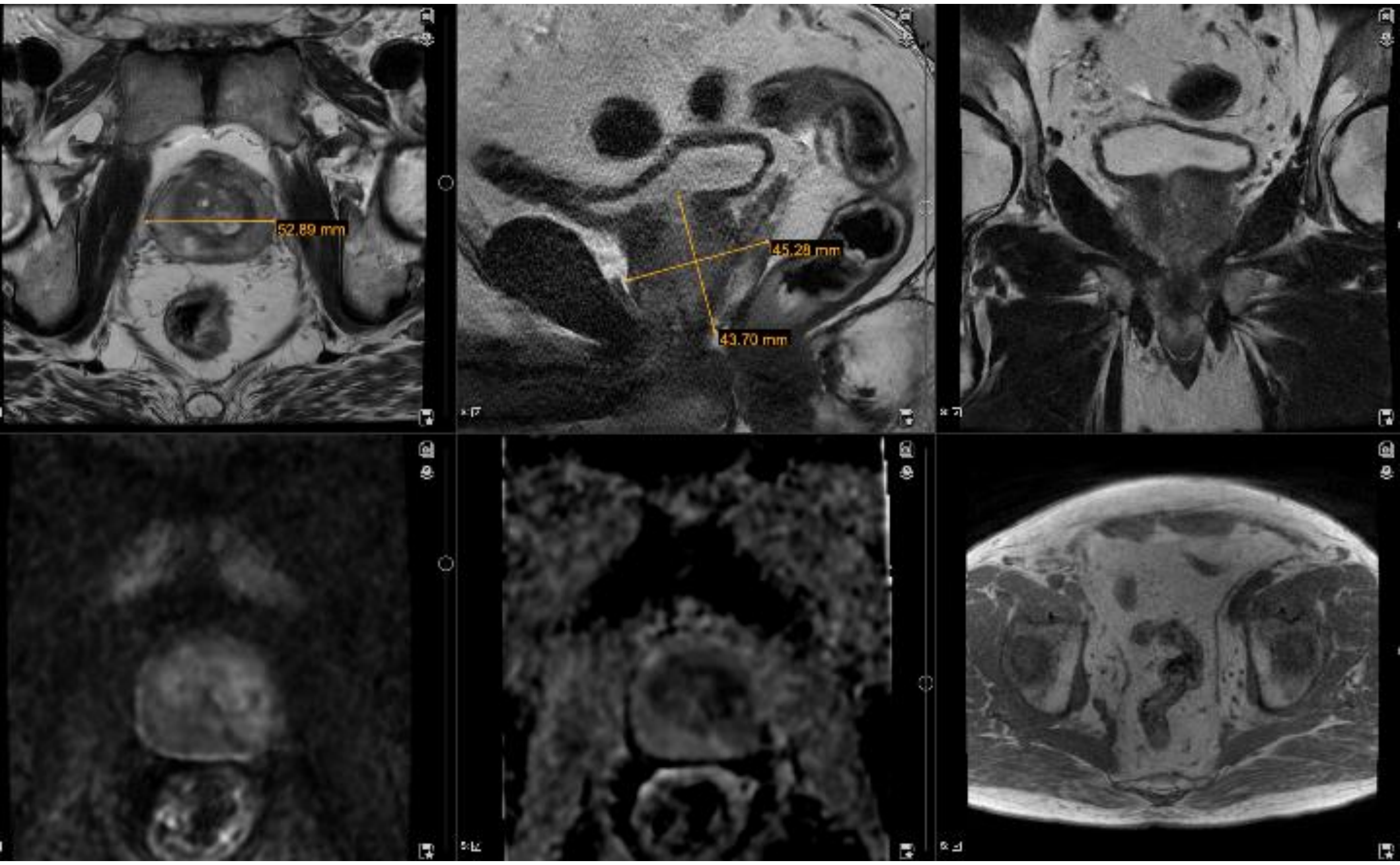

Volume measurement example.
